# Cardiomyocyte-specific deletion of the mitochondrial transporter Abcb10 causes cardiac dysfunction via lysosomal-mediated ferroptosis

**DOI:** 10.1042/BSR20231992

**Published:** 2024-05-10

**Authors:** Yura Do, Mikako Yagi, Haruka Hirai, Kenji Miki, Yukina Fukahori, Daiki Setoyama, Masatatsu Yamamoto, Tatsuhiko Furukawa, Yuya Kunisaki, Dongchon Kang, Takeshi Uchiumi

**Affiliations:** 1 Department of Clinical Chemistry and Laboratory Medicine, Graduate School of Medical Sciences, Kyushu University, Higashi-ku, Fukuoka 812-8582, Japan; 2 Department of Health Sciences, Graduate School of Medical Sciences, Kyushu University, Higashi-ku, Fukuoka 812-8582, Japan; 3 Department of Molecular Oncology, Graduate School Medical and Dental Sciences, Kagoshima University, 8-35-1 Sakuragaoka, Kagoshima 890-8544, Japan; 4 Department of Pathology, Graduate School Medical and Dental Sciences, Kagoshima University, 8-35-1 Sakuragaoka, Kagoshima 890-8544, Japan

**Keywords:** heart failure, lysosomes, mitochondria

## Abstract

Heart function is highly dependent on mitochondria, which not only produce energy but also regulate many cellular functions. Therefore, mitochondria are important therapeutic targets in heart failure. Abcb10 is a member of the ABC transporter superfamily located in the inner mitochondrial membrane and plays an important role in haemoglobin synthesis, biliverdin transport, antioxidant stress, and stabilization of the iron transporter mitoferrin-1. However, the mechanisms underlying the impairment of mitochondrial transporters in the heart remain poorly understood. Here, we generated mice with cardiomyocyte-specific loss of Abcb10. The Abcb10 knockouts exhibited progressive worsening of cardiac fibrosis, increased cardiovascular risk markers and mitochondrial structural abnormalities, suggesting that the pathology of heart failure is related to mitochondrial dysfunction. As the mitochondrial dysfunction was observed early but mildly, other factors were considered. We then observed increased Hif1α expression, decreased NAD synthase expression, and reduced NAD^+^ levels, leading to lysosomal dysfunction. Analysis of ABCB10 knockdown HeLa cells revealed accumulation of Fe^2+^ and lipid peroxides in lysosomes, leading to ferroptosis. Lipid peroxidation was suppressed by treatment with iron chelators, suggesting that lysosomal iron accumulation is involved in ferroptosis. We also observed that Abcb10 knockout cardiomyocytes exhibited increased ROS production, iron accumulation, and lysosomal hypertrophy. Our findings suggest that Abcb10 is required for the maintenance of cardiac function and reveal a novel pathophysiology of chronic heart failure related to lysosomal function and ferroptosis.

## Introduction

Mitochondria are dynamic organelles responsible for a variety of cellular functions, including energy production, iron metabolism, fatty acid and amino acid oxidation, and apoptosis [1]. Mitochondrial cardiomyopathy is a common manifestation of mitochondrial respiratory failure and is associated with dilated cardiomyopathy and heart failure [2]. Moreover, structural damage to mitochondria and mitochondrial dysfunction occur in heart failure, including increased mitochondrial oxidative damage, impaired mitochondrial respiration, and abnormal utilization of mitochondrial substrates [3].

Many transport proteins are expressed in mitochondria, which help mitochondria to maintain homeostasis via the release of various substances and interactions with other organelles. ABCB10, a member of the ABC transporter superfamily found in the inner membrane of human mitochondria, forms homodimers that are oriented with the ATP-binding domains facing toward the mitochondrial matrix [4]. ABCB10 is expressed at particularly high levels in the mitochondria of blood cell progenitors and is primarily expressed in bone marrow, liver, and heart. Constitutive knockout of *Abcb10* in mice produced severely anemic embryos at 10.5 days post coitum and higher levels of apoptosis in erythroid precursors, suggesting that ABCB10 is required for erythropoiesis [5]. Heterozygous *Abcb10* mice also display impaired recovery from ischemia–reperfusion injury because of the increased production of reactive oxygen species (ROS) [6].

ABCB10 functions by interacting with mitoferrin 1 (MFRN1) and ferrochelatase to promote heme synthesis and, together with MFRN1, is responsible for iron uptake into erythrocyte mitochondria. Reduced ABCB10 expression leads to decreased MFRN1 protein levels and impaired iron import into mitochondria, reducing heme synthesis [7,8]. Mice with hematopoietic tissue-specific inducible deletion of *Abcb10* (driven by Mx1-Cre) showed increased iron deposits in mitochondria and accumulation of protoporphyrin IX in immature erythrocytes. These results provide new insights into both heme biosynthesis and our understanding of the pathogenesis and treatment of protoporphyria and sideroblastic anemia [9]. In addition, a recent study using an Abcb10-reconstituted liposome system revealed biliverdin as a substrate of Abcb10, with hepatic *Abcb10* deletion in mice leading to the accumulation of biliverdin in mitochondria [10]. However, the function of Abcb10 in the heart remains unclear.

Although caspase-dependent apoptosis has long been considered the primary pathway of cardiomyocyte death, ferroptosis has recently been shown to play an important role in the pathogenesis of cardiovascular disease [11]. In a recent report regarding doxorubicin (DOX)-induced cardiomyopathy, mitochondria-dependent ferroptosis was found to be a major cause of DOX induced cardiotoxicity [12]. Tadokoro et al. showed that DOX down-regulated the levels of glutathione peroxidase 4 (GPX4), a major regulator of ferroptosis, and induced excessive lipid peroxidation via the DOX-Fe^2+^ complex in mitochondria. In another study, Fang et al. found that excess free iron accumulated in mitochondria, leading to the generation of lipid peroxides in mitochondrial membranes. MitoTEMPO, a superoxide scavenger designed to target the mitochondria, rescued DOX-induced cardiomyopathy and exerted cardioprotective effects against ferroptosis-induced heart damage [13]. Additionally, in a rat model of acute heart failure caused by transverse aortic constriction (via aortic banding), Chen et al. demonstrated the role of TLR4 and NADPH oxidase 4 (NOX4) in autophagy and ferroptosis. *Gpx4* and *Fth1* expression levels were reduced in the aortic banding control but restored in groups with knockdown of either TLR4 or NOX4. Moreover, GPX4-dependent ferroptosis in this model suggested TLR4-NOX4 as a potential therapeutic target for heart failure [14,15].

To examine the potential role of Abcb10 in chronic heart failure, we generated a cardiac myocyte-specific *Abcb10* knockout mouse. Examination of these mice revealed that Abcb10 KO induced heart failure and mitochondrial dysfunction, as well as lysosomal dysfunction leading to ferroptosis. Moreover, Abcb10 knockout mice died at around the age of 12 months. These results provide new insights into the pathogenesis of chronic heart failure. To the best of our knowledge, this is the first report to show the effects of Abcb10 deficiency not only on cardiac mitochondrial function but also on lysosomal function and ferroptosis in the heart.

## Methods

### Animals

All animal experiments took place at the Animal center of Kyushu University. Animal care was conducted in compliance with Kyushu University Animal Care Guidelines (#A23-233). All experimental procedures conformed to the Guide for the Care and Use of Laboratory Animals, 8th Edition, updated by the US National Research Council Committee in 2011. The animals were treated in accordance with the guidelines stipulated by Kyushu University Animal Care and Use Committee. *Abcb10^flox/flox^* mice were obtained from the laboratory of Yamamoto and Furukawa at Kagoshima University [9], and mice expressing a Cre recombinase under the cardiac-specific alpha myosin-heavy chain (*αMHC*) Myh6 promoter were obtained from Jackson Laboratories. To generate mice with heart-specific knockout of *Abcb10*, we crossed *Abcb10^flox/flox^* mice with *αMHC-cre* (Myh6 promoter) mice [9,16]. Mice at different stages of disease were anesthetized with an overdose of sevoflurane. After exsanguination under deep anaesthesia, the mice were dissected and used for the respective experiments as previously reported [17].

### Cell culture

HeLa cells were cultured in Dulbecco’s modified Eagle’s medium (DMEM; 1000 mg/L glucose; Sigma-Aldrich) supplemented with 10% fetal bovine serum at 37°C in a humidified atmosphere with 5% CO_2_. Cell were incubated with the Deferoxamine mesylate (DFO) salt powder, ≥92.5% (TLC), (100 μM; Sigma-Aldrich, 205-314-3) for 3 h.

### ROS generation

Mitochondrial superoxide produces were detected by MitoSOX Red (absorption/emission maxima of ∼396/610 nm) (Thermo Fisher, Massachusetts, U.S.A.). Approximately 500 nM MitoSOX solution was added to ardiomyocyte and incubated for 30 min at 37°C in 5% CO_2_. Fluorescence imaging of the MitoSOX-stained cells via microscopy were measured fluorescence intensity.

### Isolated adult cardiomyocyte

WT and Abcb10 cKO mice aged 7–9 months were anesthetized, and immediately the heart was transferred to a 60-mm dish containing fresh EDTA buffer (130 mM NaCl, 5 mM KCl, 0.5 mM NaH_2_PO_4_, 10 mM HEPES, 10 mM glucose, 10 mM butanedione monoxime, 10 mM taurine, 5 mM EDTA). Digestion was achieved by collagenase buffer (130 mM NaCl, 5 mM KCl, 0.5 mM NaH_2_PO_4_, 10 mM HEPES, 10 mM glucose, 10 mM butanedione monoxime, 10 mM taurine, 1 mM MgSO_4_, 50 μg/ml Collagenase (FUJIFILM Wako, Osaka, Japan)) into the left ventricle. Cellular dissociation was completed by gentle trituration, and enzyme activity was inhibited by addition of 5% FBS. Cell suspension was passed through a 100-μm filter, and cells underwent gravity settling. The cell pellet formed a highly pure myocyte fraction. The cardiac nonmyocyte fraction was collected by centrifugation (300 ***g*** for 5 min), resuspended in DMEM, and cultured on collagen-coated dishes [18]. WT cardiomyocytes were incubated with Erastin (10 μM, Sigma-Aldrich), Ferrostatin-1 (5 μM, Sigma-Aldrich) for 24, 48, and 72 h.

### Immunohistochemistry of heart sections

After mice were anesthetized with an overdose of sevoflurane, the hearts were fixed in 10% formaldehyde and paraffin-embedded. Tissue sections (coronal) were prepared and stained with various antibodies. Argon laser light (488 and 540 nm) was used to excite lipofuscin autofluorescence. TrueBlack™ (Biotium, California, U.S.A.) reagents were used before primary antibody treatment [16].

### Oxygen consumption rate assay

Mitochondria were extracted from the heart as follows. The tissue was crushed in isolation buffer (215 mM mannitol, 75 mM sucrose, 1 mM EGTA, 20 mM HEPES, pH7.4), sonicated, and centrifuged at 3,000 rpm for 10 min at 4°C to remove unbroken tissues and nuclei. The supernatant was further centrifuged at 10,000 rpm for 10 min at 4°C to enrich the mitochondria. The extracted mitochondria were adjusted to a concentration of 1 µg/µl by BCA protein assay and suspended in reaction buffer (215 mM mannitol, 75 mM sucrose, 20 mM HEPES pH7.4, 2 mM MgCl_2_, 2.5 mM KH_2_PO_4_). Oxygen consumption rates were measured using a Seahorse XFe24 Analyzer (Agilent, California, U.S.A.) under basal conditions or following the addition of 1 mM ADP, 10 µM oligomycin, 4 µM FCCP (uncoupler), and 1 µM rotenone/antimycin A (electron transport inhibitor), in accordance with the manufacturer’s protocol.

### Knockdown of ABCB10 in HeLa cells

For *ABCB10* knockdown, siRNA (Sigma-Aldrich) was transfected using Lipofectamine RNAiMAX (Thermo Fisher) in accordance with the manufacturer’s instructions.

### RNA extraction

Immediately after dissection, heart tissue was immersed in RNAlater (Invitrogen, CA, U.S.A.) overnight, and then RNA was extracted. The ReliaPrep™ RNA Miniprep System (Promega, Wisconsin, U.S.A.) was used for RNA extraction from heart tissue and the RNeasy Mini Kit (QIAGEN, Venlo, The Netherlands) was used for the extraction of RNA from cells (after using a QIAshredder [Qiagen] to homogenize the cells). cDNA was synthesized using total RNA (500 ng), random hexamer primers, oligo dT primers, and the PrimeScript™ RT Reagent Kit (TaKaRa, Kyoto, Japan) [19].

### Real-time PCR analysis

Total RNA was extracted using the ReliaPrep™ RNA Tissue Miniprep System (Promega). cDNA was synthesized using total RNA, random hexamer primers, oligo dT primers, and a PrimeScript™ RT reagent kit (Takara). The cDNA was then subjected to real-time PCR analysis using TB Green™ Premix Ex Taq™II (Takara) and the StepOnePlus Real-Time PCR system (Applied Biosystems). Ribosomal 18S rRNA was evaluated as an internal control [20]. Primer sequences are listed in Supplementary Table S1.

### Immunoblotting

Heart tissues were immediately frozen in liquid nitrogen. Tissues and cultured cells were lysed with RIPA buffer (50 mM Tris-HCl, pH 8.0, 150 mM NaCl, 0.5% sodium deoxycholate, 1% NP-40, 0.1% SDS, protease inhibitor cocktail [Wako, Hiroshima, Japan]), homogenized by sonication, and then subjected to immunoblotting. Protein quantities were adjusted to 5 μg/lane using the BCA protein assay kit (Nacalai Tesque, Kyoto, Japan). Adjusted samples were separated using 8%, 10%, 12%, or 15% SDS-PAGE gels and transferred to PVDF membranes. The blocking buffer used was Blocking One (Nacalai Tesque), and primary and secondary antibodies were diluted in Can Get Signal (Toyobo, Osaka, Japan). All antibody dilutions were 1:5000. Chemi-Lumi ImmunoStar® LD (FUJIFILM) was used for detection. Antibodies are listed in Supplementary Table S2.

### Electron microscopy

For electron microscopy, samples were fixed with 2% paraformaldehyde (PFA) and 2% glutaraldehyde (GA) in 0.1M phosphate buffer (PB), pH 7.4, at 4°C overnight. The method details have previously been reported [21].

### Fluorescence probes

Detection of intracellular Fe^2+^ was performed using FerroOrange (excitation, 561 nm; emission, 570–620 nm) (DOJINDO, Kumamoto, Japan). Cells were cultured in glass-bottomed dishes for 3 days and washed with HBSS (Thermo Fisher), after which 1 µM FerroOrange was added for 30 min at 37°C, followed by observation under a microscope (BZ-X800; KEYENCE). To detect Fe^2+^ in mitochondria, 5 µM Mito-FerroGreen working solution (excitation, 488 nm; emission, 500–550 nm) (DOJINDO, Kumamoto, Japan) was added to the cells for 30 min at 37°C, followed by observation under a microscope. Lipid radicals were detected using LipiRADICAL Green (Funakoshi, Tokyo, Japan) (excitation, 470 nm; emission, 520–600 nm) and Liperfluo (excitation, 524 nm; emission, 535 nm) (DOJINDO, Kumamoto, Japan). Cells and cardiomyocyte were cultured in glass-bottomed dishes for 3 days and washed with HBSS, after which 1 µM LipiRADICAL Green or 4 µM Liperfluo working solution was added for 10 min at 37°C, followed by observation under a microscope (BZ-X800; KEYENCE).

### GSH/GSSG assay

GSSG/GSH Quantification Kit was used to measure the amount of GSH and GSSG, which are Glutathione peroxidase 4 (Gpx4) substrates (DOJINDO, Kumamoto, Japan), according to the supplier’s instructions.

### Metabolome assay LC-MS/MS

Heart-derived metabolites were analyzed by LC-MS/MS based on reverse-phase ion-pair chromatography and hydrophilic interaction chromatography modes coupled with a triple quadrupole mass spectrometer, LCMS-8040 (Shimadzu, Kyoto, Japan). For the monitoring of metabolites, including intermediates in central metabolism, reverse-phase ion-pair chromatography was performed using an ACQUITY UPLC BEH C18 column (100 × 2.1 mm, 1.7 µm particle size; Waters). The mobile phase consisted of solvent A (15 mM acetic acid and 10 mM tributylamine) and solvent B (methanol), and the column oven temperature was 40°C. The gradient elution program was as follows: a flow rate of 0.3 ml/min: 0–3 min, 0% B; 3–5 min, 0–40% B; 5–7 min, 40–100% B; 7–10 min, 100% B; and 10.1–14 min, 0% B. Parameters for negative electrospray ionization mode (ESI) under multiple reaction monitoring (MRM) were as follows: drying gas flow rate, 15 L/min; nebulizer gas flow rate, 3 L/min; DL temperature, 250°C; heat block temperature, 400°C; and collision energy, 230 kPa. Meanwhile, for monitoring 61 kinds of metabolites including amino acids, hydrophilic interaction chromatography was performed using a Luna 3u HILIC 200A column (150 × 2 mm, 3 µm particle size; Phenomenex). The mobile phase consisted of solvent A (10 mM ammonium formate in water) and solvent B (9:1 of acetonitrile:10 mM ammonium formate in water), and the column oven temperature was 40°C. The gradient elution program was as follows: a flow rate of 0.3 ml/min: 0–2.5 min, 100% B; 2.5–4 min, 100–50% B; 4–7.5 min, 50–5% B; 7.5–10 min, 5% B; and 10.1–12.5 min, 100% B. Parameters for positive and negative ESI mode under MRM were as described above. Data processing was performed using LabSolutions LC-MS software program (Shimadzu) [21].

### Lysosome fraction

Mouse hearts were homogenized in a BioMasher tube (Nippi) containing HB buffer (250 mM sucrose, 10 mM HEPES-KOH, 1mM EDTA, proteinase inhibitor cocktail). Samples were then centrifuged at 3000 rpm for 10 min at 4°C. The supernatant was centrifuged at 10,000 rpm for 3 min at 4°C, and protein from the supernatant was then adjusted to 3 μg using the BCA protein assay kit (Nacalai Tesque, Kyoto Japan). The sample adjusted to 3 μg was centrifuged at 15,000 rpm for 30 min at 4°C. The pellet was used for the lysosome fraction and then subjected to immunoblotting.

### Mitochondrial fraction

Mouse hearts were homogenized in a BioMasher tube (Nippi) containing HB buffer (250 mM sucrose, 10mM HEPES-KOH, 1mM EDTA, proteinase inhibitor cocktail). Samples were then centrifuged at 3000 rpm for 10min at 4°C. The protein from supernatant was adjusted to 5 mg using the BCA protein assay kit (Nacalai Tesque, Kyoto Japan). The sample adjusted to 5 mg was centrifuged at 10,000 rpm for 6 min at 4°C. The pellet was used for mitochondrial fraction and then subjected to analyzing by LC-MS/MS for biliverdin analysis.

### Quantification and statistical analysis

The data displayed represent at least three independent experiments *in vitro*. Mice used for the *in vivo* experiments were randomly selected. All Western blotting experiments were quantified using Las 4000 and statistical analysis in Prism8. Immunostaining brightness was quantified with BZ-X800 and statistical analyses with Prim8.

## Results

### Cardiac myocyte-specific Abcb10 knockout causes cardiac dysfunction

**Figure 1:**
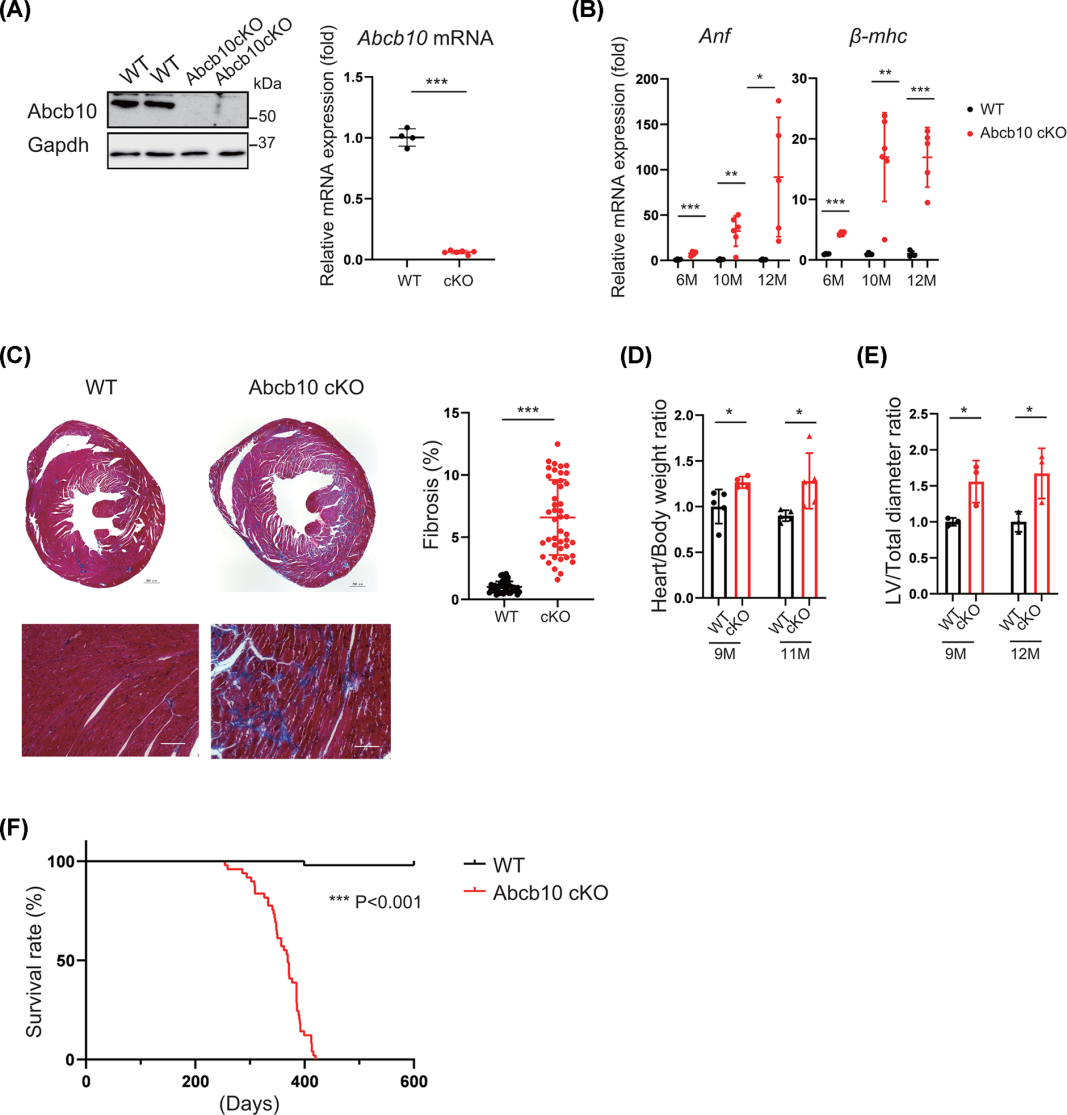
Abcb10 deletion in mouse cardiomyocytes causes cardiac dysfunction and shortened lifespan (**A**) Western blot and real-time qPCR analysis of Abcb10/*Abcb10* expression in WT and Abcb10cKO hearts at 6 months. Gapdh was used as internal control (WT, *n*=4; Abcb10 cKO, *n*=6). (**B**) Relative mRNA expression of cardiac failure markers *Anf* and *β-mhc* in WT and Abcb10cKO mouse hearts at 6, 10, and 12 months of age (WT, *n*=4–6; Abcb10 cKO, *n*=5–6). (**C**) Representative images of Masson's trichrome staining of heart sections from WT and Abcb10 cKOs at 10 months. Fibrosis in hearts was quantified by measuring the blue staining per tissue area (6 sections per sample). Scale bars for whole heart sections = 500 µm; scale bars for sections below = 100 µm. (WT, Abcb10 cKO: *n*=4). (**D**) Heart weight-to-body weight ratios of 9- and 11-month-old mice (WT, *n*=5; Abcb10 cKO, *n*=4). (**E**) Left ventricular diameter to total diameter ratio of ventricle mid-region heart cross sections (WT, Abcb10 cKO: *n*=3). (**F**) Survival curve for male WT and Abcb10 cKO mice (WT male: *n*=17, female:* n*=31; Abcb10 cKO male: *n*=18, female: *n*=31). In A–E, error bars are presented as mean ±SD. Statistical significance was assessed using the Student’s *t*-test; *P<0.05, **P<0.01, ***P<0.001.

To investigate the role of Abcb10 in cardiac function, we crossed mice bearing the *Abcb10 ^flox^* allele with *αMHC-Cre* mice and generated cardiac-specific Abcb10-deficient mice. Analysis of *Abcb10* expression in the hearts of Abcb10 conditional knockout (cKO) mice confirmed reduced levels of transcripts and protein (Figure 1A). We also found that the levels of myocardial *atrial natriuretic factor* (*Anf*) and *β-myosin heavy chain* (*β-mhc*) mRNAs were significantly higher in Abcb10 cKO hearts than in wild-type (WT) hearts (Figure 1B), suggesting cardiac dysfunction. Knockout mice had more severe cardiac fibrosis and a higher heart weight/body weight ratio at 11 months of age (Figure 1C,D). Furthermore, myocardial thickness was increased in Abcb10 cKO compared with WT (Figure 1E). Therefore, cardiac function was impaired in Abcb10 cKO heart.

We also evaluated the survival rate of Abcb10 cKO mice (WT, *n*=48; Abcb10cKO, *n*=49). These male and female mice died suddenly from the age of 11 months onwards, with a median lifespan of around 12 months (Figure 1F). These results indicate that ablation of cardiac Abcb10 gradually causes cardiac dysfunction and shortened lifespan, suggesting that Abcb10 is required to maintain cardiac function and therefore for survival.

### Deletion of Abcb10 induces mitochondrial dysfunction

**Figure 2:**
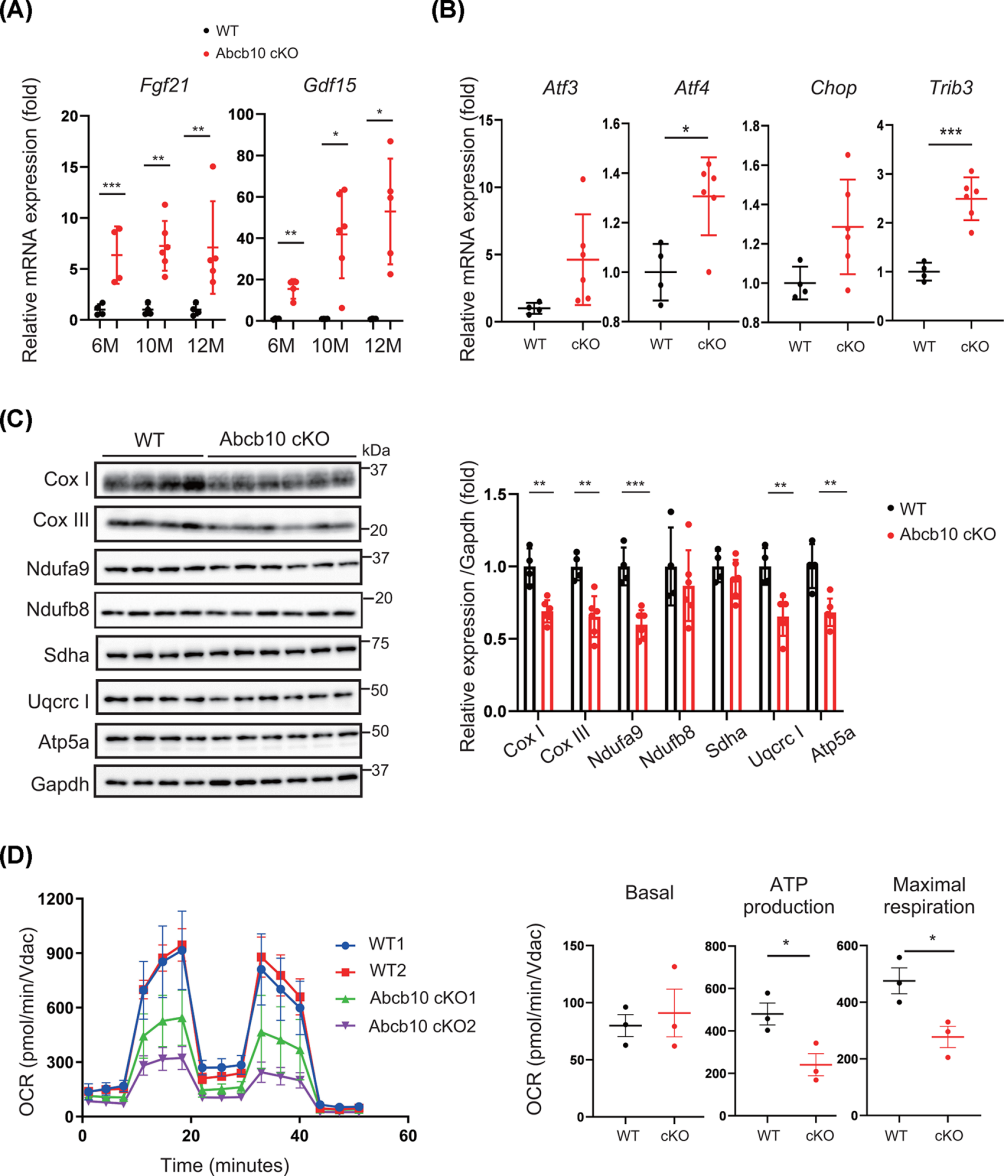
Functional characteristics of mitochondria in Abcb10 cKO hearts (**A**) Quantitative real-time qPCR expression analysis of *Fgf21* and *Gdf15* (biomarkers for mitochondrial disorders) in the hearts of 6- to 12-month-old WT and Abcb10 cKO mice (WTs, *n*=4–6; Abcb10 cKOs, *n*=5–6). (**B**) Quantitative real-time qPCR expression of integrated stress gene (*Atf3, Atf4, Chop* and *Trib3*) in heart from 10-month-old Abcb10 cKO and WT mice (WT: *n*=4, Abcb10cKO: *n*=6). (**C**) Western analysis of OXPHOS proteins in the hearts of 10-month-old WT and Abcb10 cKO mice. CoxI and CoxIII (mitochondrial DNA encoded, Complex IV), Ndufa9 and Ndufb8 (complex I), Sdha (complex II), Uqcrc1 (complex III) Atp5a (complexV). GAPDH was used as an internal control (WT, *n*=4; Abcb10 cKO, *n*=6). (**D**) Mitochondrial oxygen consumption rates (OCRs) in mitochondrial fractions from WT and Abcb10 cKO hearts from 8 months old. Results represent mean ± SD (WT, Abcb10 cKO, *n*=3). In A–D, error bars are presented as mean ±SD. Statistical significance was assessed using the Student’s *t*-test; *P<0.05, **P<0.01, ***P<0.001.

Next, we investigated mitochondrial function and structure. In the Abcb10 cKO mice, the gene expression of the mitochondrial disease biomarkers *Gdf15* (*growth differentiated factor 15)* and *Fgf21(fibroblast growth factor 21)* gradually increased with age (Figure 2A), suggesting that mitochondrial dysfunction became progressively more severe as these mice aged. We also performed integrated stress response (ISR) gene expression profiling to investigate possible disease-causing processes [22]. Real-time PCR analysis showed that, in Abcb10 cKO hearts, as in control mice with disrupted mitochondrial homeostasis (*n*=6), the expression of many ISR-activated genes (e.g., *Atf3*, *Atf4*, *Ddit3*/*Chop*, and *Trib3*) was increased (Figure 2B). These results suggest that Abcb10 knockouts exhibit a stress response due to mitochondrial dysfunction.

Analysis of the levels of Cox I and Cox III proteins, which are encoded by mitochondrial DNA, revealed significant reduction in the hearts of Abcb10 cKO mice, compared with WT mice, at 10 months of age. In addition, Abcb10 cKO mouse hearts exhibited lower levels of Ndufa9 (a subunit of complex I of the respiratory chain), as well as Uqcrc1 (complexes III) and Atp5a (complex V) (Figure 2C). Ndufb8 (Complex I) and Sdha (Complex II) were not altered in Abcb10 knockout heart. We also observed lower levels of mitochondria DNA encoded mRNAs and tRNA such as *Nd1, Nd2 and 16S* rRNA in Abcb10 cKO hearts than in controls (Supplementary Figure S1A). These results suggest that mitochondrial transcription may be affected in Abcb10 cKO hearts. To evaluate the functional effects of mitochondrial impairment, we compared the oxygen consumption rates of mitochondria from WT and Abcb10 cKO hearts. The results indicated that deletion of Abcb10 in the heart led to significant decreases in ATP production and maximal respiration rate (Figure 2D).

**Figure 3:**
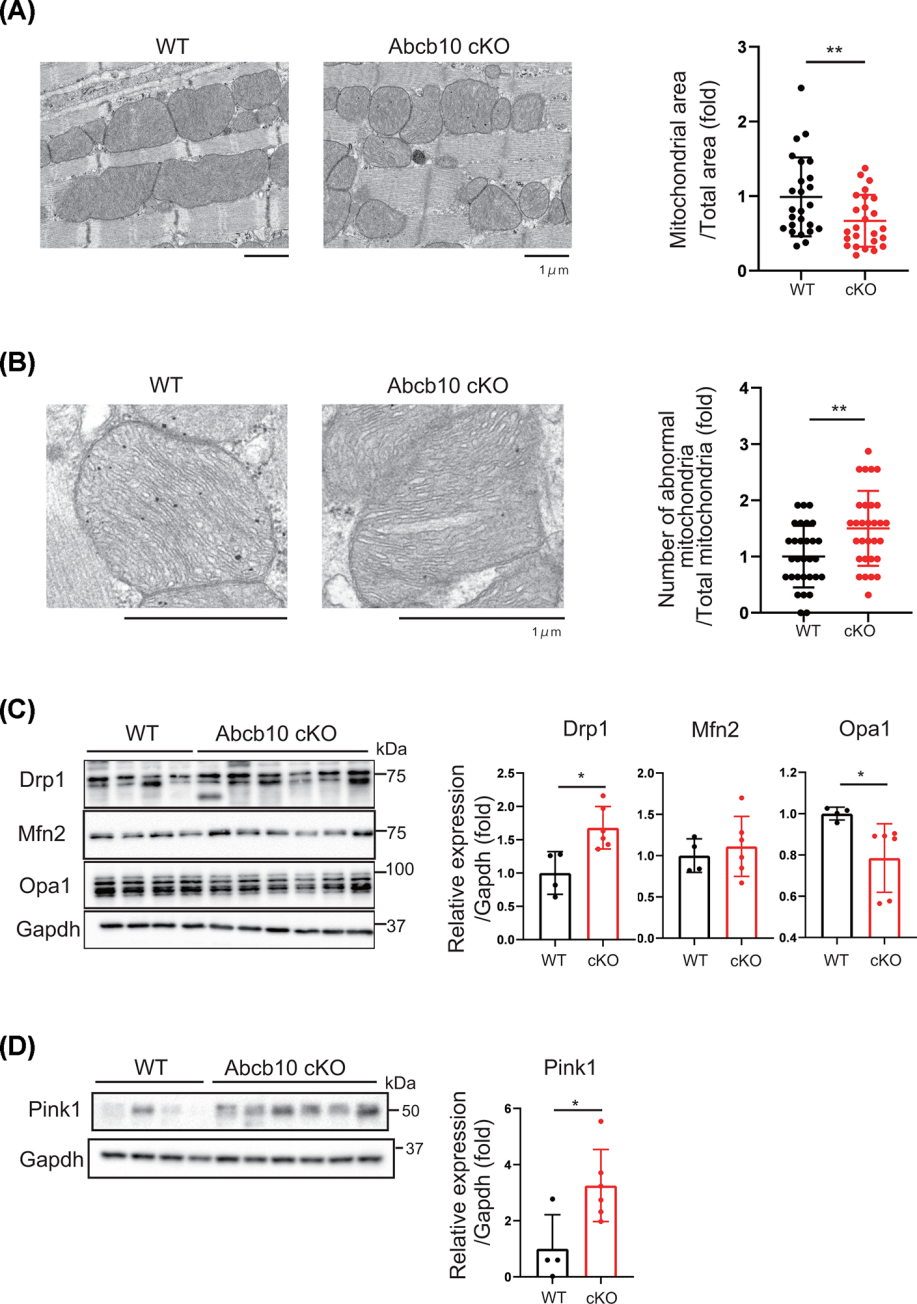
Structural characteristics of mitochondria in Abcb10 cKO hearts (**A**) Representative electron microscopy images of the arrangement and area of mitochondria in heart tissue from 10-month-old WT and Abcb10 cKO mice. Quantification was performed by determining mitochondria area/total area per sheet (5–9 sheets per group); scale bars, 1 µm. (**B**) Representative electron microscopy images of heart tissue from 10-month-old WT and Abcb10 cKO mice and quantification of abnormal mitochondria (30 sheets per group). (**C**) Western analysis of mitochondrial fission and fusion proteins in hearts of Abcb10 WT and cKO mice from 10-month-old. Gapdh was used as an internal control (WT: *n*=4, Abcb10 cKO: *n*=6). (**D**) Western analysis of mitochondrial autophagy proteins Pink1 in hearts of WT and Abcb10 cKO mice from 10-month-old. (WT: *n*=4, Abcb10 cKO: *n*=6). In **A-D**, error bars are presented as mean ± SD. Statistical significance was assessed using the Student’s *t*-test; *P<0.05, **P<0.01.

Analysis of mitochondrial ultrastructure by electron microscopy confirmed the presence of morphological modifications in Abcb10 cKO hearts. In WT hearts, normal mitochondria were found to be aligned along actin/myosin filaments, while the Abcb10 cKO hearts mitochondria were varied in size (Figure 3A and Supplementary Figure S1B). We also observed abnormal mitochondria containing vacuoles in the matrix, with loss of matrix density and apparent destruction of mitochondrial membranes in Abcb10 cKO (Figure 3B and Supplementary Figure S1B).

Mitochondrial morphological dynamics is associated with the regulation of cellular function and disease, and fragmented mitochondria are observed in cells in cases of mitochondrial disorders. To investigate the link between mitochondrial morphology and modification of mitochondrial dynamics, we measured fission or fusion proteins. We observed increased levels of fission protein Drp1 and reduced levels of fusion protein Opa1 in Abcb10 cKO hearts (Figure 3C), suggesting that mitochondria are shrinking and that damaged mitochondria are moving towards mitophagy. We next investigated whether Pink1-mediated mitophagy is occurring in Abcb10 knockout hearts. Pink1 expression was predominantly increased in Abcb10 knockout hearts, suggesting that Pink1 may accumulate in Abcb10 cKO due to mitochondrial dysfunction. (Figure 3D).

Mitochondrial morphology was then observed by Mitotracker staining. Mitochondria were classified as dot-shaped mitochondria or filamentous according to their morphology (Supplementary Figure S1C). Meanwhile, more than 60% of ABCB10 knockdown cells were classified as dot shaped mitochondria, suggesting mitochondrial dysfunction. We also observed that ABCB10 knockdown cells showed the reduced CoxI and CoxIII expression in HeLa cells (Supplementary Figure S1D).

Taken together, these ultrastructural and functional data suggest that Abcb10 is required to maintain normal mitochondrial structure and OXPHOS function, and that accumulation of abnormal mitochondria leads to cardiomyocyte dysfunction. Since mitochondrial dysfunction was observed early but was mild and persisted for up to a year, damage to other organelles besides the mitochondria was investigated.

### NAD^+^ biosynthesis is reduced in Abcb10 cKO hearts

**Figure 4:**
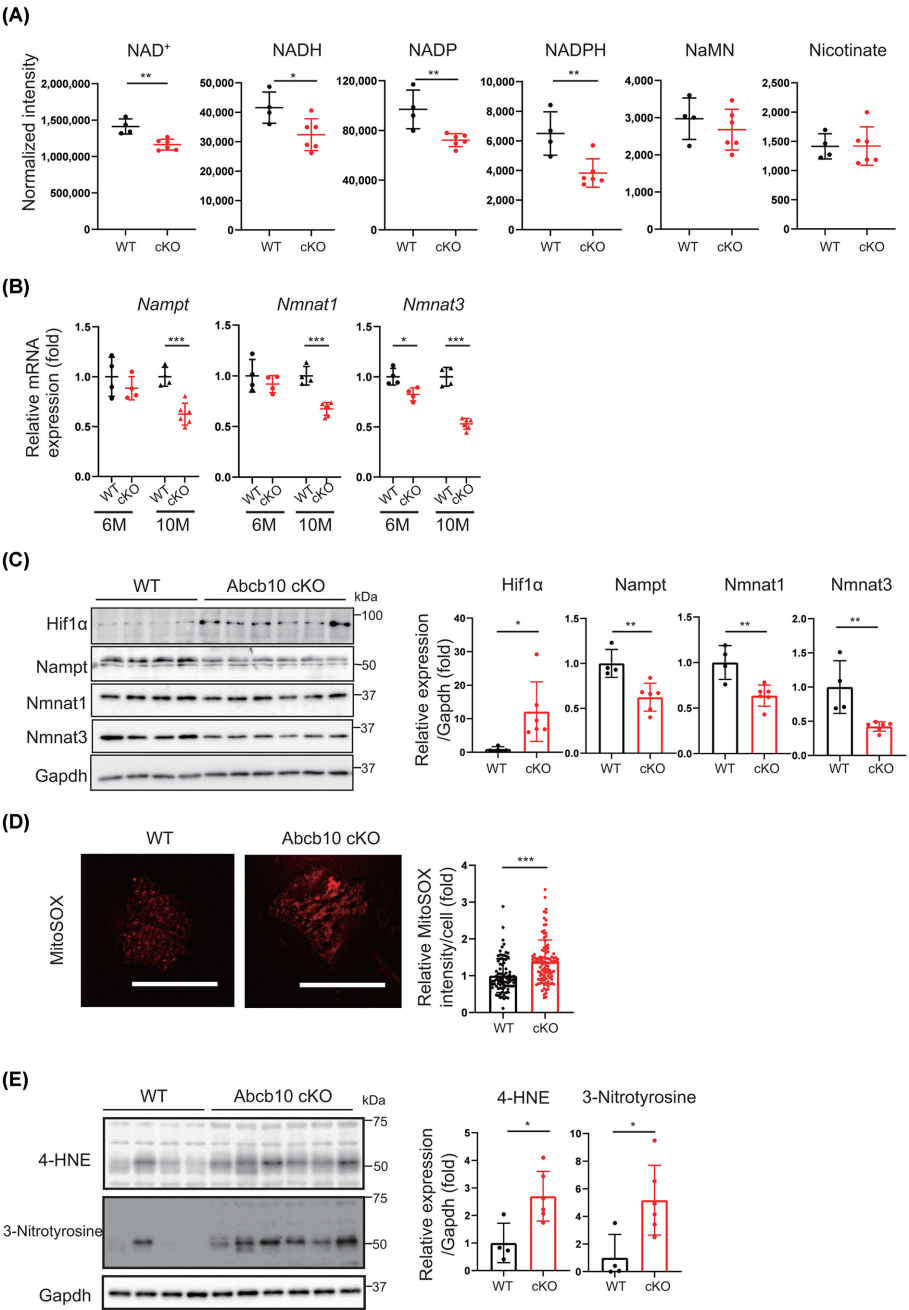
Reduced NAD biosynthesis and NAD^+^ levels in Abcb10 cKO hearts (**A**) LC-MS/MS metabolic analysis of nicotinamide adenine dinucleotide (NAD^+^), nicotinamide adenine dinucleotide, reduced form (NADH), nicotinamide adenine dinucleotide monophosphate (NADP), nicotinamide adenine dinucleotide monophosphate, reduced form (NADPH), nicotinamide mononucleotide (NaMN), and nicotinate in hearts from10-month-old WT and Abcb10 cKO mice (WT, *n*=4; Abcb10 cKO, *n*=6). (**B**) Quantitation of *Nampt*, *Nmnat1*, and *Nmnat3* mRNA levels in hearts from 6- to 10-month-old WT and Abcb10 cKO mice, measured by real time qPCR (WT, *n*=4; Abcb10 cKO, *n*=4–6). (**C**) Western blotting of NAD-synthesizing enzymes and Hif-1α in hearts from 10-month-old WT and Abcb10 cKO mice. Gapdh was used as an internal control (WT, *n*=4; Abcb10 cKO, *n*=6). (**D**) ROS production by MitoSOX fluorescent probe from WT and Abcb10 knockout cardiomyocyte. Twenty cardiomyocytes were measured per mouse heart. Scale bar, 50 µm (WT, *n*=3; Abcb10 cKO, *n* =3). (**E**) Relative protein levels of oxidative stress protein 4-HNE and 3-nitrotyrosine (WT, *n*=4; Abcb10 cKO, *n*=6). In A–E, error bars are presented as mean ±SD. Statistical significance was assessed using the Student’s *t*-test; *P<0.05, **P<0.01, ***P<0.001.

NAD^+^ is involved in pathways essential for cell survival, such as transcriptional regulation, energy metabolism, DNA repair, and inflammatory responses. Chronic dysregulation of NAD^+^-dependent cellular functions ultimately leads to the development of cardiovascular disease [23]. We performed metabolomic analysis of NAD-related energy sources by LS-MS/MS in WT and Abcb10 cKO hearts at 10 months (Figure 4A and Supplementary Figure S2). We found that the levels of NAD^+^, NADH, NADP^+^, and NADPH were significantly reduced in Abcb10 cKO hearts, and the amounts of nicotinate and nicotinic acid adenine mononucleotide remained unchanged (Figure 4A). Reduced NAD^+^ levels could have been caused by decreased NAD^+^ synthesis or increased NAD^+^ consumption. Accordingly, we examined the gene expression of NAD^+^ synthesis enzymes and found that the mRNA levels of *Nmnat1, Nmnat3*, and *Nampt*, which encode salvage pathway enzymes, were decreased in Abcb10 cKO hearts (Figure 4B). Nampt, Nmnat1, and Nmnat3 protein levels were also decreased (Figure 4C). These findings suggested that the reduced NAD^+^ content resulted from down-regulated expression of NAD^+^ synthesis genes. We have also reported that the expression of Nmnat3 is negatively regulated by the transcription factor Hif1α [16]. Notably, here, Hif1α levels were significantly upregulated in Abcb10 cKO hearts (Figure 4C), suggesting that increased expression of Hif1α, decreased expression of the *Nmnat3* gene and reduced NAD^+^ levels in Abcb10 cKO hearts.

The generation of ROS during angiogenin II treatment has been reported to be essential for both increased translation and stability of Hif1α in the vasculature [24]. To investigate whether mitochondrial ROS affect Hif1α stability in Abcb10 knockout hearts, mitochondrial ROS were measured in cardiomyocytes isolated from mouse hearts using MitoSOX, a mitochondrial-targeted probe for detecting ROS. It was found that Abcb10 knockout cardiomyocytes had significantly increased mtROS generation compared with WT cardiomyocytes (Figure 4D), which may have led to increased Hif1α.

To investigate the potential effects of Abcb10 knockout on oxidative stress, we carried out Western blotting analysis of 4-hydroxy-2-nonenal (4-HNE) and 3-nitrotyrosine-modified proteins. Staining of 4-HNE and 3-nitrotyrosine was elevated in Abcb10 cKO, compared with controls, suggesting increased oxidative stress in Abcb10-deficient mouse hearts (Figure 4E). Increased oxidative stress could have resulted from the accumulation of abnormal mitochondria, leading to cardiomyocyte dysfunction.

Metabolomic analysis of components of the tricarboxylic acid (TCA) cycle showed that isocitrate, succinate and malate levels were reduced in Abcb10 cKO mice compared with their levels in WT mice (Supplementary Figure S2A). Amino acids such as aspartic acid, leucine and methionine were increased in Abcb10 cKO mice, while arginine was decreased (Supplementary Figure S2B). These results suggest that loss of Abcb10 affects the TCA cycle and amino acid metabolism. The major antioxidant glutathione and its oxidant, GSSG, were measured in Abcb10 knockout hearts. As a result, increased GSSG levels were observed in Abcb10 knockout hearts, suggesting increased oxidative stress. (Supplementary Figure S2C).

Abcb10 has been reported to have the ability to excrete biliverdin from the mitochondria to cytosol of liver tissue. We extracted whole heart tissue and mitochondria from heart tissue and analyzed biliverdin levels by LC-MS/MS. Biliverdin levels were increased in Abcb10 cKO compared with WT in the whole heart tissue, but were unchanged in mitochondrial extracts (Supplementary Figure S2D). These results suggested that Abcb10 was involved in biliverdin levels in heart tissue.

### Cardiomyocyte-specific knockout of Abcb10 leads to the accumulation of impaired lysosomes

**Figure 5:**
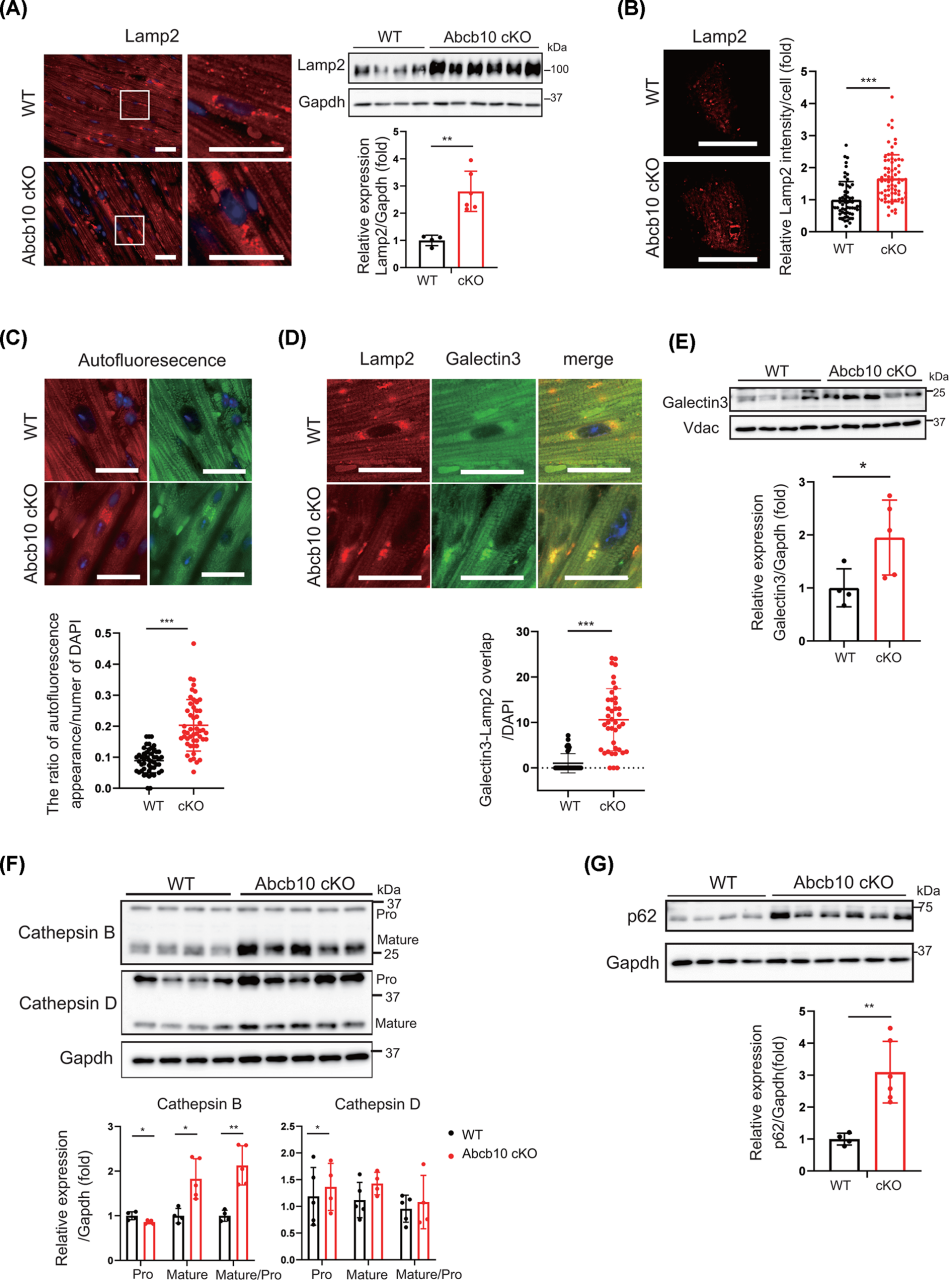
Cardiac-specific Abcb10 deficiency causes impaired lysosomal function and accumulation of p62 in hearts (**A**) Immunostaining of Lamp 2 (red) in heart sections with DAPI-stained nuclei (blue). Western blot showing increased Lamp 2 in 10-month-old WT and Abcb10 cKO hearts. Gapdh was used as an internal control. Error bars are presented as means ± SD (WT, *n*=4; Abcb10 cKO: *n*=6). (**B**) Immunostaining of Lamp2 in cardiomyocyte from each mouse heart. Relative Lamp2 intensity was measured in twenty cardiomyocyte from each mouse heart; scale bar, 50 µm. Error bars are presented as means ± SD (WT: *n*=3, Abcb10 cKO: *n*=3). (**C**) Detection of lipofuscin granules around the nuclei by autofluorescence in heart sections. Tissues were excited at a wavelength of 540 or 470 nm, and emission spectra were collected using a confocal microscope at wavelengths of 580–630 nm or 510–560 nm. Quantification of the ratio of autofluorescence appearance per DAPI staining area is presented in the right panel; scale bar, 20 µm. Error bars are presented as means ± SEM (WT and Abcb10 cKO, *n* =4; 12 sheets per group). (**D**) Immunostaining of Lamp 2 (red) and Galectin 3 (green) in 10-month-old WT and Abcb10 cKO hearts; scale bar, 20 µm. Quantification on the right shows the increased co-localization of Lamp 2 and galectin 3 in Abcb10 cKO hearts. Error bars are presented as means ± SD (WT and Abcb10 cKO, *n*=4; 10 sheets per group). (**E**) Western blot showing increased Galectin3 protein level in lysosomal fraction of 11-month-old WT and Abcb10 cKO heart. Vdac, located in the mitochondrial outer membrane, is used as loading control (WT: *n*=4, Abcb10 cKO: *n*=5). (**F**) Western blotting of Capthepsin B, Cathepsin D and Gapdh in 10-month-old WT, Abcb10 cKO hearts (WT: *n*=4, Abcb10 cKO: *n*=6). (**G**) The accumulation of autophagic marker protein p62 in 10-month-old Abcb10 cKO hearts. Gapdh was used as an internal control. Error bars are presented as means ± SD (WT, *n*=4; Abcb10 cKO, *n*=6).

Previously, we found that mitochondrial translation-deficient hearts from p32-knockout mice showed impaired lysosomes as a result of reduced NAD content [16]. Thus, we investigated lysosomal function in Abcb10-deficient hearts. Lysosomal dysfunction has been reported to lead to substrate accumulation, resulting in hypertrophy and migration of lysosomes to the perinuclear area [25]. Here, the levels of the lysosome-associated glycoprotein Lamp2 were shown to be increased in Abcb10 cKO hearts (Figure 5A); in particular, immunostaining showed numerous large, dot-like structures localized around the nuclei in Abcb10 cKO heart tissue, which were absent in WT heart tissue (Figure 5A). Structures such as Lamp2 dots and increased fluorescence intensity, indicating increased Lamp2 protein, were also observed in Abcb10 knockout cardiomyocytes (Figure 5B). These results suggest that lysosomal dysfunction occurs in Abcb10 knockout cardiomyocytes.

In cardiac tissue, lipofuscin is known to accumulate with heart failure and age. Lipofuscin consists of highly autofluorescent granules of oxidized proteins and lipids that accumulate in the lysosomes of aging cells. To assess lysosomal function in cardiac tissue, we detected autofluorescence in heart sections from Abcb10 cKO and WT using microscopy. In WT hearts, there was little autofluorescence around the nuclei, whereas in Abcb10 cKO hearts, much lipofuscin appeared as multiple fluorescent granules around the nuclei (Figure 5C).

We also examined the expression and localization of galectin 3, which is involved in membrane repair, removal, and replacement and is known as a marker of damaged lysosomes [26]. Cardiac tissue sections from Abcb10 cKO mice were co-stained for Lamp2 (red) and galectin 3 (green). Abcb10 cKO hearts showed numerous damaged lysosomes around the nuclei, manifesting as yellow dots of co-localized Lamp2 and galectin 3 proteins; by contrast, in WT hearts, galectin 3 was not observed in such aggregates (Figure 5D). Moreover, the levels of galectin 3 were increased 2-fold in Abcb10 cKO mice hearts, compared with levels in WT hearts (Figure 5E). These findings indicated increased numbers of damaged lysosomes in Abcb10 cKO hearts.

Next, to investigate the degradative capacity of the lysosomes, we examined the expression of the intra-lysosomal protease cathepsin B and cathepsin D. The protein levels of mature cathepsin B showed an increasing trend in Abcb10 cKO hearts, compared with WT hearts (Figure 5F). However, the expression of cathepsin D was not significantly increased. Taken together, these results suggest that the accumulation of mature cathepsin B and structurally abnormal lysosomes in Abcb10 cKO hearts, leading to lysosomal abnormality.

### p62 accumulates in Abcb10 cKO hearts

Increased autophagy in the heart is implicated in heart failure [27]. Therefore, we investigated whether lysosomal dysfunction in Abcb10 cKO hearts occurs as a result of impaired autophagy. The autophagy marker p62 plays important roles in initiating autophagy and recruiting ubiquitinated proteins and organelles to autophagosomes for degradation. To investigate the potential impairment of autophagy activation in Abcb10 cKO hearts, we examined p62 expression. We found that the levels of p62 protein were significantly increased in Abcb10 cKO hearts (Figure 5G). In addition, immunostaining revealed many rings of p62 in Abcb10 cKO hearts, which were not observed in WT heart (Supplementary Figure S3A). Another autophagosomal marker, LC3-II, is generated by the conjugation of LC3-I to phosphatidylethanolamine on the surface of autophagosomes. In heart tissue from 12-month-old Abcb10 cKOs, the protein expression of LC3-I was increased (Supplementary Figure S3B). Taken together, our results suggest a lack of autophagic degradation and failure to resolve autophagosomal membranes as a result of the decreased degradative capacity of lysosomes.

It was reported that skeletal muscles of aged mice displayed decreased autophagic activity as reflected by the reduction in the conjugation of LC3-I with PE (phosphatidylethanolamine) upon decreased expression of Atg12-Atg5 and Atg3 protein levels [28]. In our Abcb10 cKO hearts, LC3-II/LC3-I was reduced, Atg5-Atg12 protein levels were unchanged and Atg3 and Atg7 levels were increased (Supplementary Figure S3C). These results suggest that autophagy function decreased but it is not known whether LC3 decreased with PE.

### Ferroptosis is activated in *Abcb10*-deleted hearts

**Figure 6:**
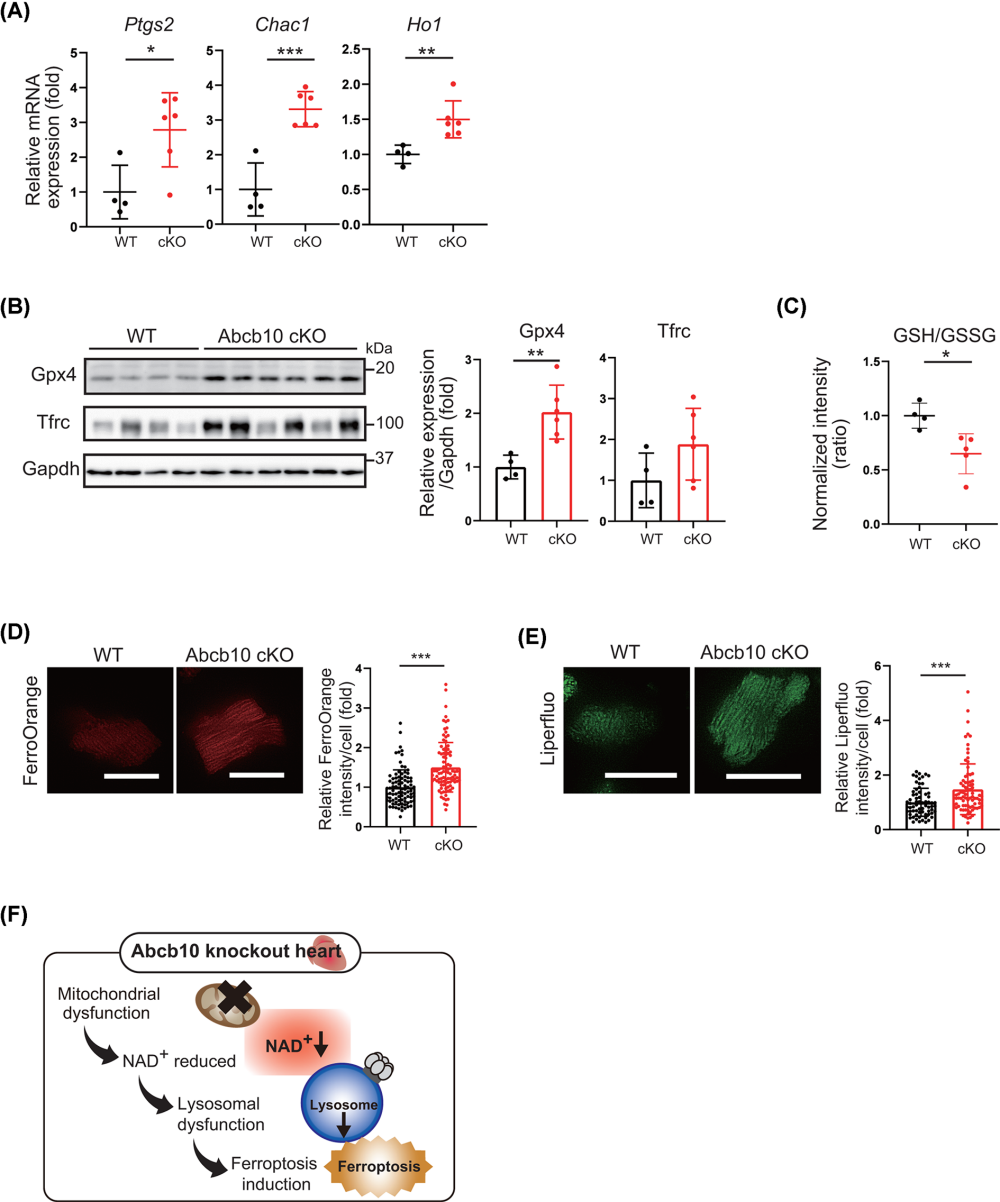
Abcb10 deficiency results in decreased GSH/GSSG ratios and induces ferroptosis (**A**) The mRNA levels of ferroptosis-related genes (*Ptgs2*, *Chac1*, and *Ho-1*) in heart tissue from 10-month-old WT and Abcb10 cKO mice were determined by real time-PCR (WT, *n*=4; Abcb10 cKO, *n* =6). (**B**) Western blot analysis of the levels of GPX4 and TFRC in 10-month-old WT and Abcb10 cKO hearts. Gapdh was used as an internal control (WT, *n*=4; Abcb10 cKO, *n*=6). (**C**) LC-MS/MS metabolomic analysis of GSH and GSSG in WT and Abcb10 cKO hearts. GSH/GSSG ratios were decreased in 10-month-old Abcb10 cKO hearts (WT, *n*=4; Abcb10 cKO, *n*=5). (**D**) Representative image of FerroOrange (cytoplasmic iron level) in cardiomyocyte from WT and Abcb10 cKO heart. Thirty cardiomyocytes from each mouse heart were measured; scale bar, 50 µm (WT and Abcb10 cKO, *n*=3). (**E**) Representative image of Liperfluo (lipidperoxide) in cardiomyocyte from WT and Abcb10 cKO heart. Forty cardiomyocytes from each mouse heart were measured; scale bar, 50 µm (WT and Abcb10 cKO, *n*=2). (**F**) Predicted pathogenesis mechanism of dilated cardiomyopathy in Abcb10 cKO mice. In A–E, error bars are presented as mean ± SD. Statistical significance was assessed using the Student’s *t*-test; *P<0.05, **P<0.01, ***P<0.001.

Ferroptosis has been found to contribute to the progression of various cardiovascular diseases. Next, we examined markers of iron metabolism and ferroptosis related to lysosomal function. The results indicated that the expression of prostaglandin-endoperoxide synthase 2 (*Ptgs2*) and ChaC glutathione-specific gamma-glutamylcyclotransferase 1 (*Chac1*), known genetic biomarkers of ferroptosis [29], was increased in Abcb10 cKO hearts at 10 months of age (Figure 6A); the expression of heme oxygenase 1 (*Ho1*), which acts as a critical mediator in ferroptosis induction [30], was also increased (Figure 6A). Moreover, Western blotting showed increased levels of the transferrin receptor (TFRC), which mediates iron uptake and is specific to ferroptosis [31,32], as well as the key ferroptosis regulator GPX4, in Abcb10 cKO hearts (Figure 6B). Abcb10 cKO and WT glutathione (GSH) and oxidised GSH (GSSG) levels were then analysed, as GSH is required for peroxidised glutathione activity and reduced GSH may lead to reduced GPX4 activity. This analysis revealed a decreased GSH-to-GSSG ratio in Abcb10 cKO hearts (Figure 6C). Together, these results suggest that GPX4 activity was reduced as a result of Cha1-mediated suppression of GSH levels.

To explore the relationship between Abcb10 deficiency and ferroptosis at the cellular level, we investigated the content and localization of iron in Abcb10 knockout cardiomyocyte. Detection of Fe^2+^ using FerroOrange (a fluorescent probe that enables live-cell fluorescent imaging of intracellular Fe^2+^) showed higher levels of fluorescence intensity in Abcb10 knockout cardiomyocyte than in WT cardiomyocyte (Figure 6D). To visualize lipid peroxidation in living cells, we used Liperfluo, a detection reagent for lipid radicals, which are upstream factors of lipid peroxidation. The Liperfluo Green fluorescent signal was significantly stronger Abcb10 knockout cardiomyocyte than WT (Figure 6E), indicating that ferroptosis has been induced in Abcb10 cKO hearts. These results suggest that Abcb10 knockout mice exhibit early mitochondrial damage and dilated cardiomyopathy, followed by reduced NAD levels that impair lysosomal and autophagic function, ultimately leading to cell damage by ferroptosis (Figure 6F).

**Figure 7:**
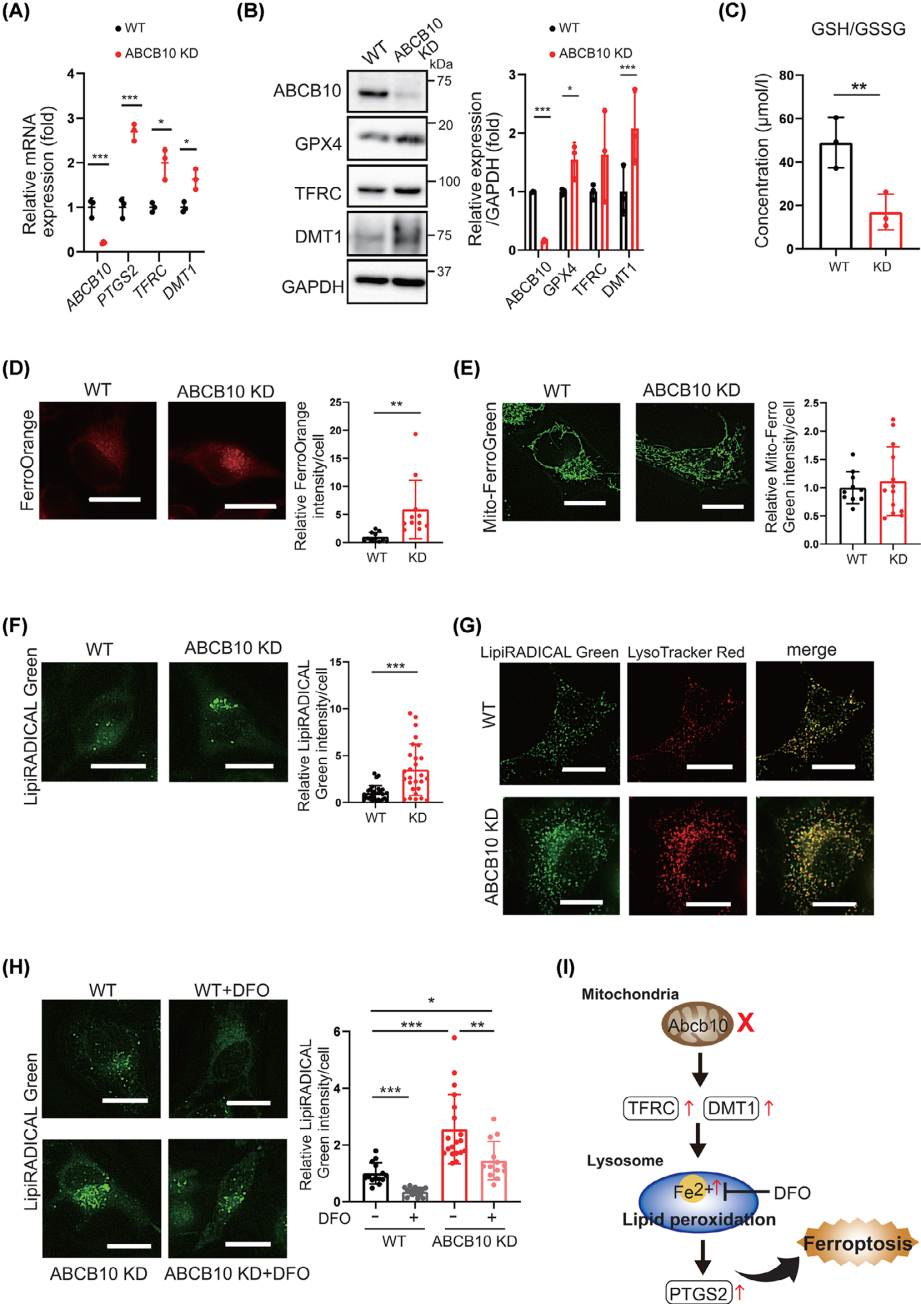
ABCB10 siRNA treatment causes intracellular iron accumulation and triggers lysosomal lipid peroxidation (**A**) The mRNA levels of ferroptosis-related genes in HeLa cells treated with ABCB10 siRNA for 72 h (*n*=3). (**B**) Western blot analysis of ferroptosis-related proteins in HeLa cells treated with ABCB10 siRNA for 72 h. GAPDH was used as an internal control (*n*=3). (**C**) Detection of intracellular GSH and GSSH concentrations in HeLa cells treated with ABCB10 siRNA for 72 h. The GSH/GSSG ratio was decreased in ABCB10 siRNA-treated cells (*n*=3). (**D**) Intracellular Fe^2+^ was detected by FerroOrange using fluorescence microscopy. The relative fluorescence intensity of FerroOrange was increased in HeLa cells treated with ABCB10 siRNA (*n*=11); scale bar: 20 µm. (**E**) Detection of mitochondrial Fe^2+^ using Mito-FerroGreen in HeLa cells treated for 72 h with ABCB10 siRNA. The Mito-FerroGreen fluorescence signals were of similar intensity in WT and ABCB10 siRNA-treated cells (*n*=11); scale bar: 20 µm. (**F**) Representative confocal images of WT cells and HeLa cells treated with ABCB10 siRNA for 72 h and stained with LipiRADICAL Green (detection reagent for lipid radicals). LipiRADICAL Green relative fluorescence intensity was increased in ABCB10 siRNA-treated cells (*n*=28); scale bar: 20 µm. (**G**) LipiRADICAL Green and LysoTracker Red were used to co-stain HeLa cells treated with ABCB10 siRNA for 72 h. Representative colocalization images of lipid peroxidation in lysosomes; scale bar: 20 µm. (**H**) Intracellular lipid radical staining by LipiRADICAL Green in HeLa cells. WT: untreated, WT+DFO: HeLa and addition of 100 μM DFO for 3 h, ABCB10 KD: ABCB10 knockdown, ABCB10+DFO: ABCB10 knockdown and addition of 100 μM DFO for 3 h. The right panel shows the intensity of LipiRADICAL Green per cell; scale bar: 20 μm (*n*=13–19). Error bars are presented as mean ± SD. One-way ANOVA with Tukey’s multiple comparisons test, *P<0.05, **P<0.01, ***P<0.001. (**I**) Schematic diagram of a potential ferroptosis pathway in Abcb10 cKO hearts. Red arrows indicate increases or decreases in Abcb10 cKO, compared with levels in WT. In A–F, error bars are presented as mean ± SD. Statistical significance was assessed using the Student’s *t*-test; *P<0.05, **P<0.01, ***P<0.001.

To further analyze the molecular mechanism of ferroptosis in ABCB10 deficiency, we investigated iron- and ferroptosis-related gene expression in ABCB10-knockdown HeLa cell as HeLa cells readily take up various fluorescent probes and easily knockdown by siRNA technology. Consistent with the results in heart tissue, we observed increased expression of the ferroptosis-related genes *PTGS2* and *TFRC* in ABCB10 knockdown cells (Figure 7A). Furthermore, Western blotting showed that the levels of DMT1, which mediates the excretion of Fe^2+^ ions from lysosomes, were increased in knockdown cells (Figure 7B). To evaluate GPX4 activity, we assessed the GSH/GSSG ratios. The results showed that the concentration of GSH versus that of GSSG was reduced in ABCB10-knockdown cells, indicating reduced GPX4 activity, as demonstrated in the heart (Figure 7C). Taken together, these findings suggest that in ABCB10-deficient myocardium and cultured cells, lipid peroxidation and ferroptosis associated with iron accumulation induced by HO1 and TFRC may be caused by decreased GPX4 activity.

### ABCB10 deficiency increases intracellular iron levels, resulting in iron accumulation in lysosomes and increased lipid peroxidation

To explore the relationship between ABCB10 deficiency and ferroptosis at the cellular level, we investigated the content and localization of iron in ABCB10 knockdown and WT HeLa cells. Detection of Fe^2+^ using FerroOrange showed higher levels of fluorescence intensity in ABCB10 knockdown cells than in WT cells (Figure 7D). We also observed that higher levels of fluorescence intensity in Abcb10 knockout cardiomyocyte than in WT cardiomyocyte (Figure 6D). To examine the site of accumulation of the increased Fe^2+^ levels, we examined lysosomal localization using LysoPrime Green (Supplementary Figure S4A) and found that most intracellular Fe^2+^ was accumulated in lysosomes. Next, we specifically examined mitochondrial Fe^2+^ levels using Mito-FerroGreen, which selectively reacts with mitochondrial Fe^2+^. The fluorescence intensity of Mito-FerroGreen was similar in WT and ABCB10-knockdown cells (Figure 7E and Supplementary Figure S4B), indicating that intracellular Fe^2+^ was increased within lysosomes in ABCB10-knockdown cells rather than within mitochondria.

To visualize lipid peroxidation in the context of living cells, we used LipiRADICAL Green, a detection reagent for lipid radicals, which are upstream factors of lipid peroxidation. The LipiRADICAL Green fluorescent signal was significantly stronger in HeLa cells transfected with ABCB10 siRNA for 72 h than in WT cells (Figure 7F). Moreover, the LipiRADICAL Green signal was mostly localized in lysosomes, but not in mitochondria (Figure 7G and Supplementary Figure S4C), indicating the accumulation of lipid radicals within lysosomes. We also found that deferoxamine (DFO) treatment, an iron chelator, suppressed the LipiRADICAL Green intensity in ABCB10-knockdown cells, suggesting that ABCB10-knockdown cells occurred iron-dependent lipid peroxidation (Figure 7H). Whether ferroptosis induces cell damage in cardiomyocytes was also investigated. Treatment of cardiomyocytes with elastin, a ferroptosis inducer, reduced cell number, whereas treatment with Fer-1, which inhibits lipid peroxidation, prevented cell death (Supplementary Figure S4D). This suggests that short-term induction of ferroptosis also causes cell damage in cardiomyocytes. These results suggest that loss of ABCB10 results in increased intracellular Fe^2+^ concentrations in lysosomes, leading to lipid peroxidation and ultimately lysosomal ferroptosis. We, therefore, propose cardiac ferroptosis resulting from lysosomal dysfunction and iron accumulation as the major pathogenic mechanism in Abcb10 myocardial-specific KO mice (Figure 7I).

## Discussion

Our study revealed the pathogenesis of cardiac dysfunction caused by deficiency of the mitochondrial transporter Abcb10. To analyze the pathophysiology and physiological role of Abcb10 in the heart, we generated myocardial-specific KO mice. These mice showed gradually deteriorating cardiac function and died prematurely at around 1 year of age, demonstrating that Abcb10 has important roles in cardioprotection and sustaining life. Cardiac function is highly dependent on mitochondria, organelles that are responsible for the regulation of various cellular functions. Here, Abcb10 knockout in the heart resulted in impaired mitochondrial function, indicated by progressively elevated expression of mitochondrial disease markers. In addition, mitochondria displayed morphological abnormalities and reduced respiratory chain activity. As Abcb10 is a transporter in the inner mitochondrial membrane, our report suggests that the impairment of mitochondrial transport function or mitochondria dysfunction in matrix causes cardiac dysfunction.

We have previously shown that mice with a cardiomyocyte-specific knockout of the mitochondrial translation factor p32 develop heart failure from dilated cardiomyopathy. Defects in mitochondrial translation cause not only mitochondrial dysfunction but also reduced levels of nicotinamide adenine dinucleotide (NAD^+^), leading to impaired lysosomal acidification and autophagy. NMN treatment restored lysosomal acidification and prolonged life span, suggesting that mitochondrial dysfunction induced cardiomyopathy, leading to reduced lysosomal damage and death [16,21,33]. In this study, a similar phenotype was observed in Abcb10 cKO hearts as in p32 cKO hearts, suggesting that a similar mechanism caused cardiomyopathy, induced lysosomal dysfunction and subsequent death. Therefore, it is possible that common lysosomal dysfunction as well as mitochondrial dysfunction caused dilated cardiomyopathy.

In the pathophysiological analysis of heart failure caused by mitochondrial dysfunction, impaired mitochondrial translation has been reported to induce stabilization of Hif1α and suppress expression of *Nmnat3*, which encodes NAD synthase; the resulting reduction in NAD^+^ levels impaired lysosomal function and caused autophagic abnormalities [16]. Furthermore, the glycolytic enzymes GAPDH and PGK1, which are associated with lysosomal vesicles, were linked to ATP production by NAD^+^. Consistent with these findings, we found increased HIF1α expression in Abcb10 cKO mouse hearts, alongside decreased levels of NAD-related enzymes. These results suggest that the reduced NAD^+^ content in Abcb10 cKO hearts may result from the decreased expression of NAD^+^ synthesis genes, regulated by Hif1α (Figure 4). Reduced intracellular NAD^+^ levels can cause increased oxidative stress and decreased ATP production via alterations in mitochondrial metabolism [34]. Importantly, as declining NAD^+^ levels are associated with many hallmarks of aging, NAD^+^ could be a potential therapeutic target for aging-related diseases [35]. Recent studies have reported therapeutic strategies to increase or supplement NAD^+^ levels via the administration of a NAD^+^ biosynthesis precursor [36]. In future studies, we will investigate whether NAD^+^ precursors improve the lifespan and cardiac function of Abcb10 cKO mice.

In the present study, we found that lysosomal dysfunction related to iron accumulation led to ferroptosis. In Abcb10 cKO hearts, we observed perinuclear localization of enlarged lysosomes with increased levels of Lamp2, lipofuscin autofluorescence, and galectin 3. Lipofuscin is formed by the intralysosomal accumulation of lipid peroxides, which damage cell membranes and induce cell death by ferroptosis. Regarding the ferroptosis pathway, we confirmed increased gene expression of ferroptosis markers *PTGS2*, *CHAC1*, and *HO1* in Abcb10 cKO hearts. Moreover, metabolite analysis showed decreased GSH/GSSG ratios in Abcb10-deficient mice, indicative of reduced GPX4 activity. GSH depletion resulting from GPX4 inactivation is an important process in ferroptosis and is implicated in cardiac dysfunction and cardiomyopathy [37]. We also found up-regulation of *Ho1*, which plays a role in ferroptosis induction, and TFRC, which transfers iron into the cell. Transferred iron is released from endosomes into the cytoplasm by DMT1. In the context of Abcb10 deficiency, impaired lysosomes were increased in number and/or failed to be degraded, presumably resulting in increased DMT1 present in lysosomal membranes. These findings suggest that lysosomal dysfunction may result in an inability to release iron and consequent iron accumulation in lysosomes, leading to lipid peroxide formation and eventually ferroptosis. Consistent with this possibility, we observed intracellular accumulation of both Fe^2+^ and lipid peroxides in lysosomes in ABCB10 knockdown HeLa cells, resulting in lysosomal ferroptosis. Therefore, from a mechanistic perspective, we propose that the pathway for ferroptosis induction in Abcb10 deficiency involves increased lipid peroxidation as a result of *PTGS2* up-regulation and iron accumulation in lysosomes.

In summary, our findings suggest that Abcb10 is required to maintain cardiac function and facilitate survival. We propose that Abcb10 cKO mice develop cardiac ferroptosis associated with lysosomal dysfunction as a model of chronic heart failure.

## Supplementary Material

Supplementary Figures S1-S4 and Tables S1-S2

## Data Availability

All data are included in this manuscript and in the supplementary data.

## Abbreviations

4-HNE: 4-hydroxy-2-nonenal
cKO: conditional knockout
DFO: deferoxamine
GSH: glutathione
TCA: tricarboxylic acid
TFRC: transferrin receptor
WT: wild-type
