# Supplementary material for: Cardiomyocyte-specific deletion of the mitochondrial transporter Abcb10 causes cardiac dysfunction via lysosomal-mediated ferroptosis
Source: Biosci Rep. 2024 May 10;44(5):BSR20231992. doi: 10.1042/BSR20231992 (PMC11088307; doi:10.1042/BSR20231992)

Full unedited gel for Figure 1

(A)

Abcb10

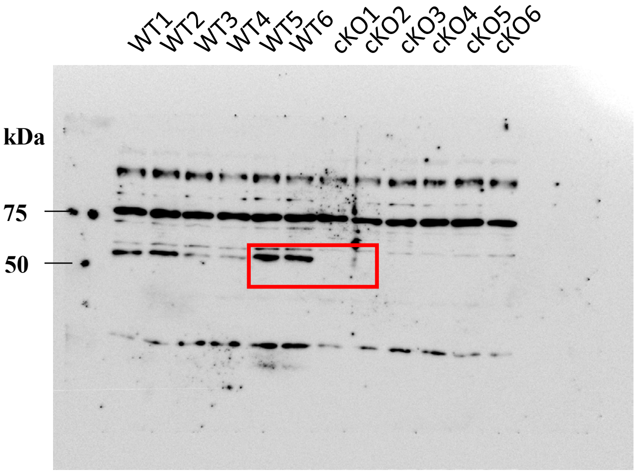

Gapdh

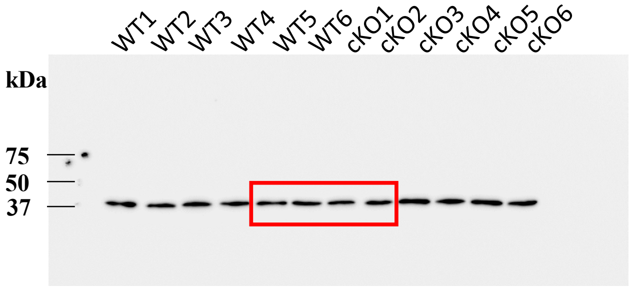

Full unedited gel for Figure 2

(C)

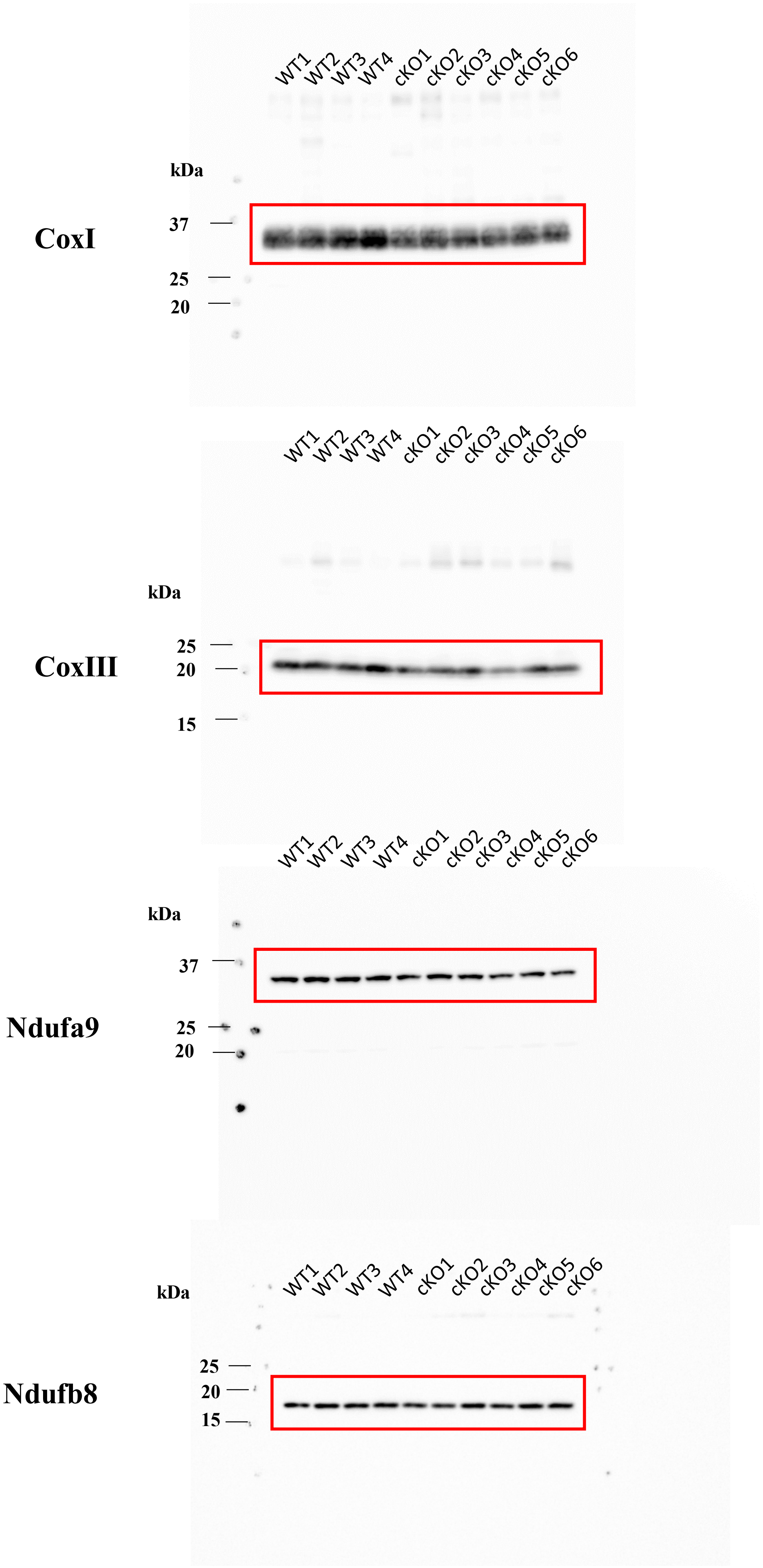

Full unedited gel for Figure 2

(C)

Sdha

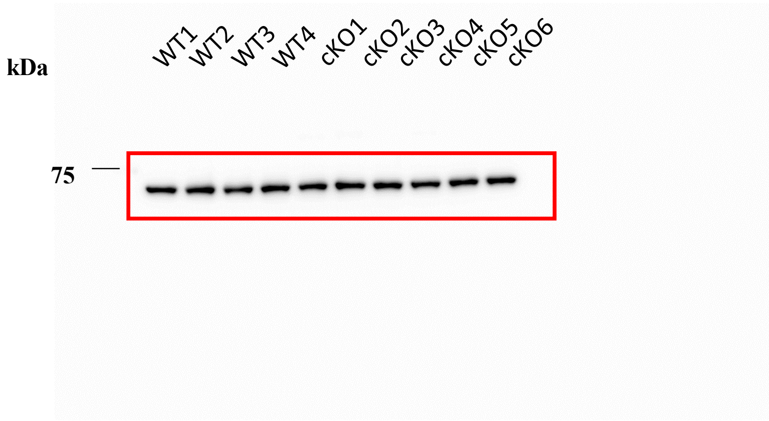

UqcrcI

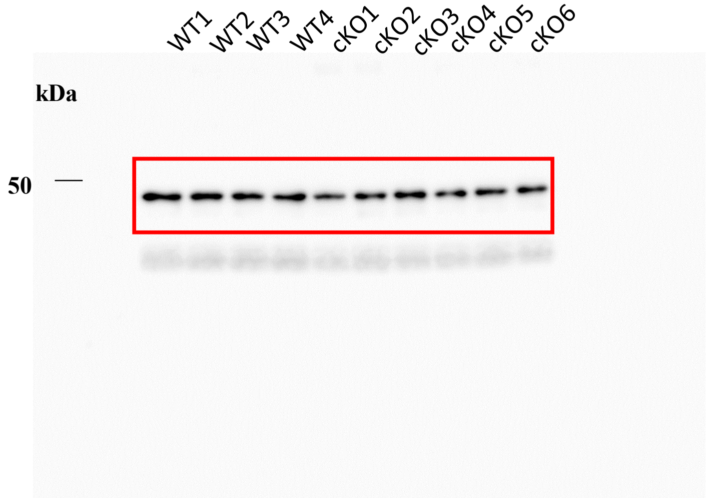

Atp5a

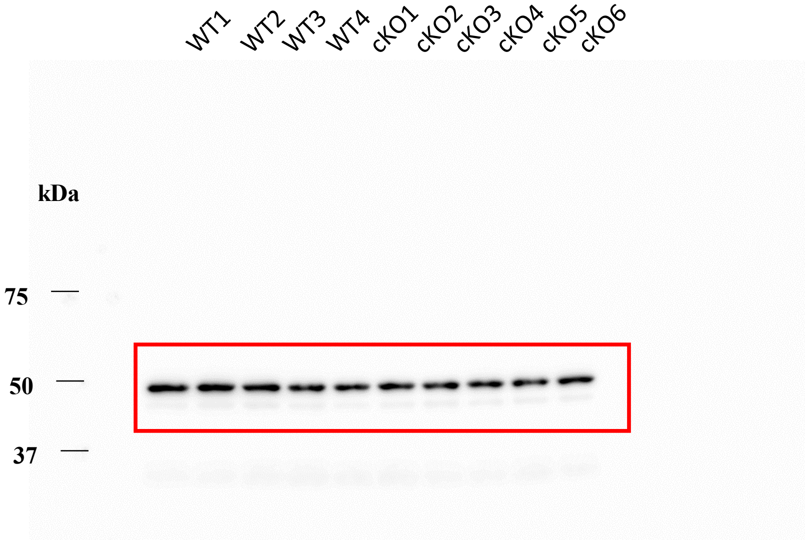

Gapdh

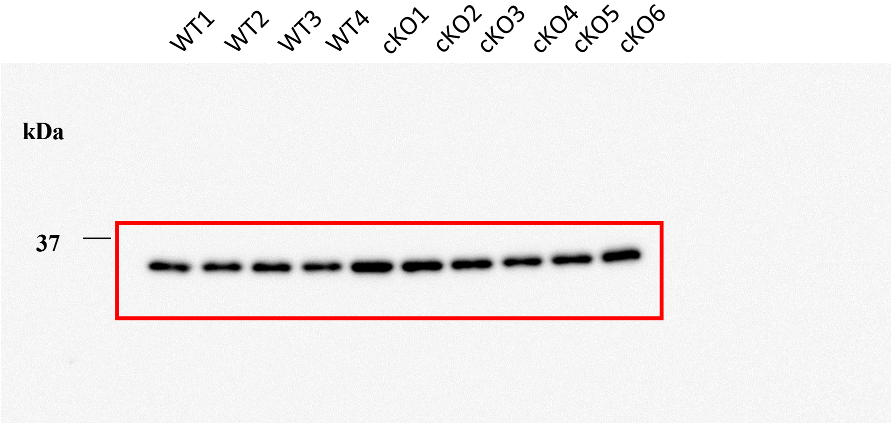

Full unedited gel for Figure 3

(C)

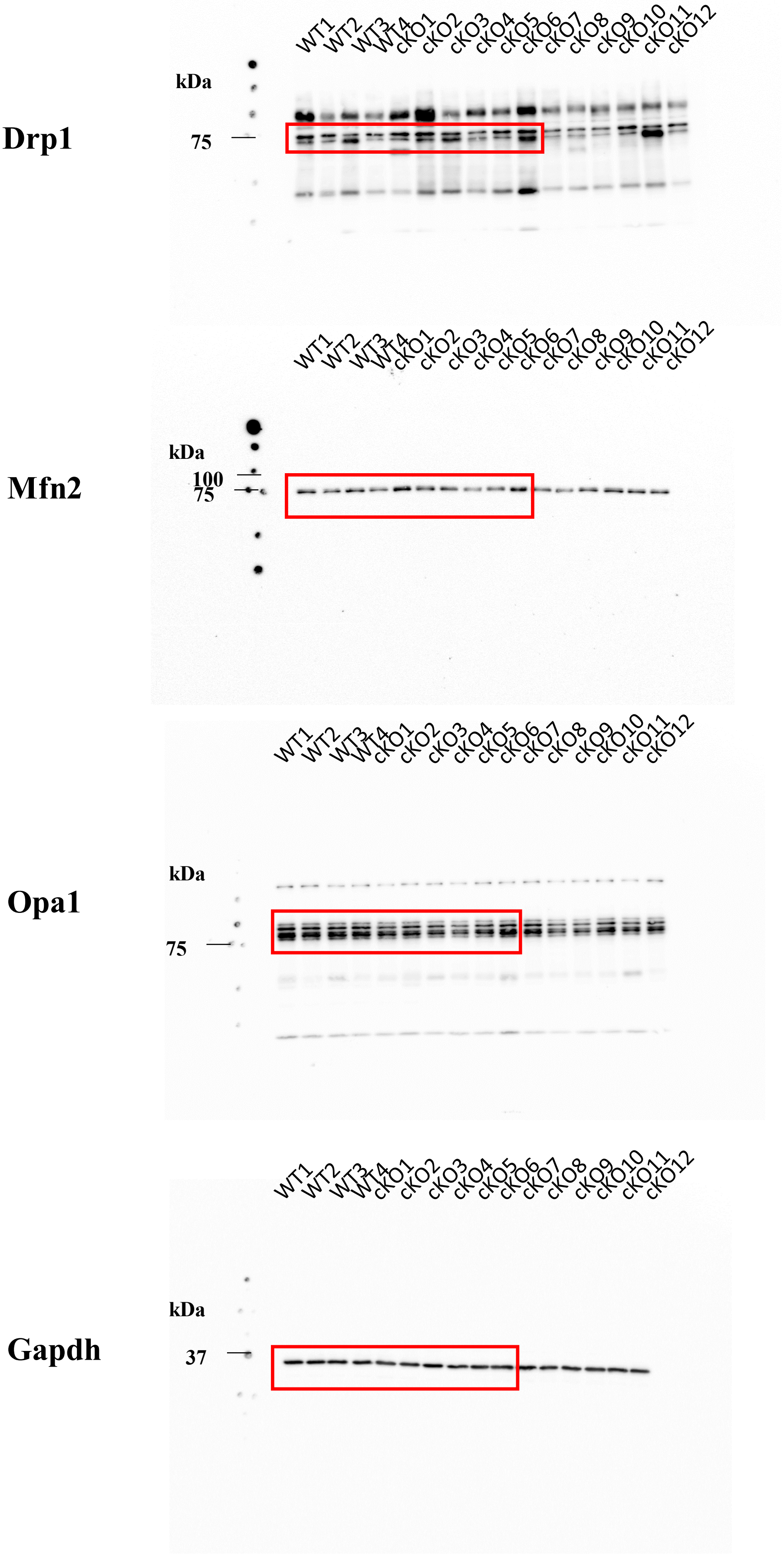

Full unedited gel for Figure 3

(D)

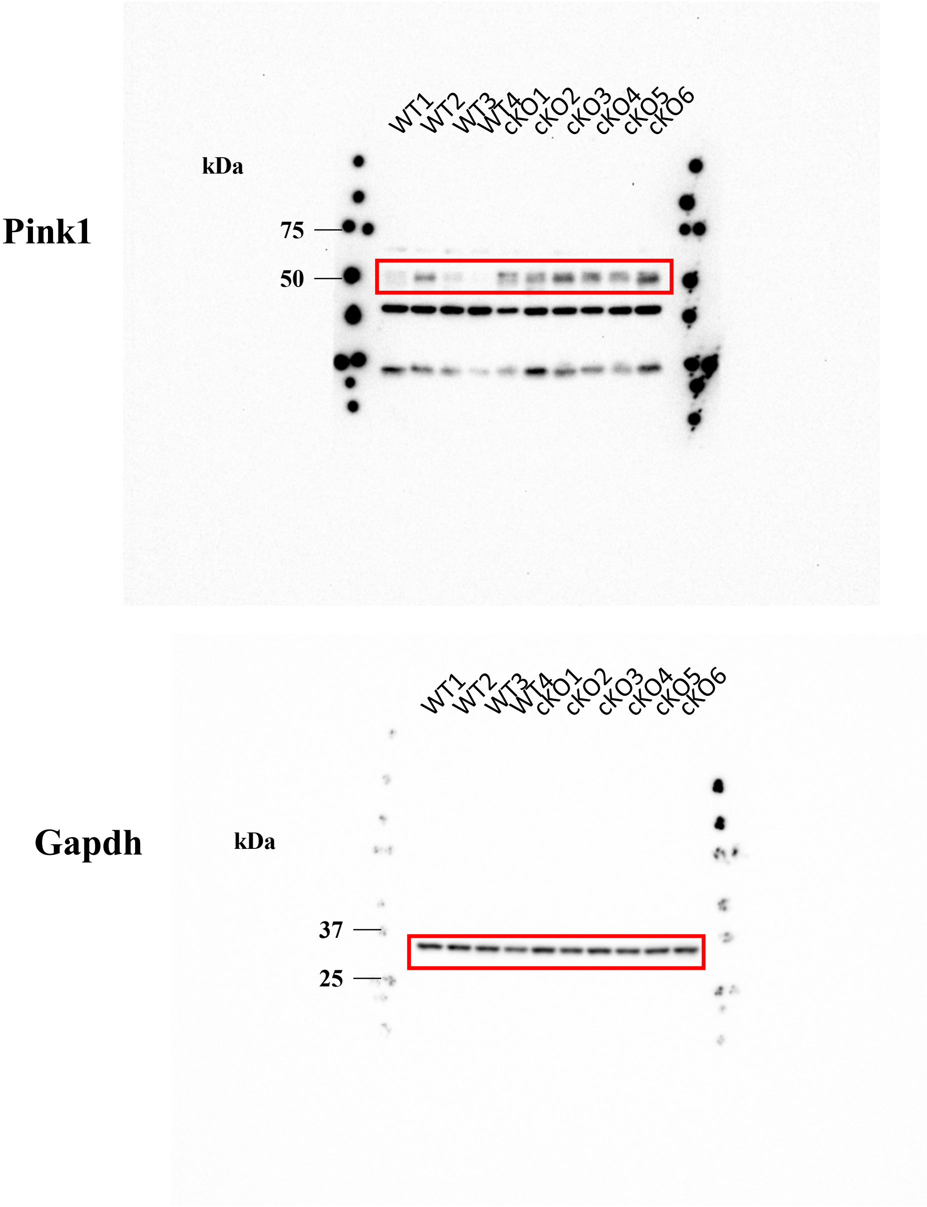

Full unedited gel for Figure 4

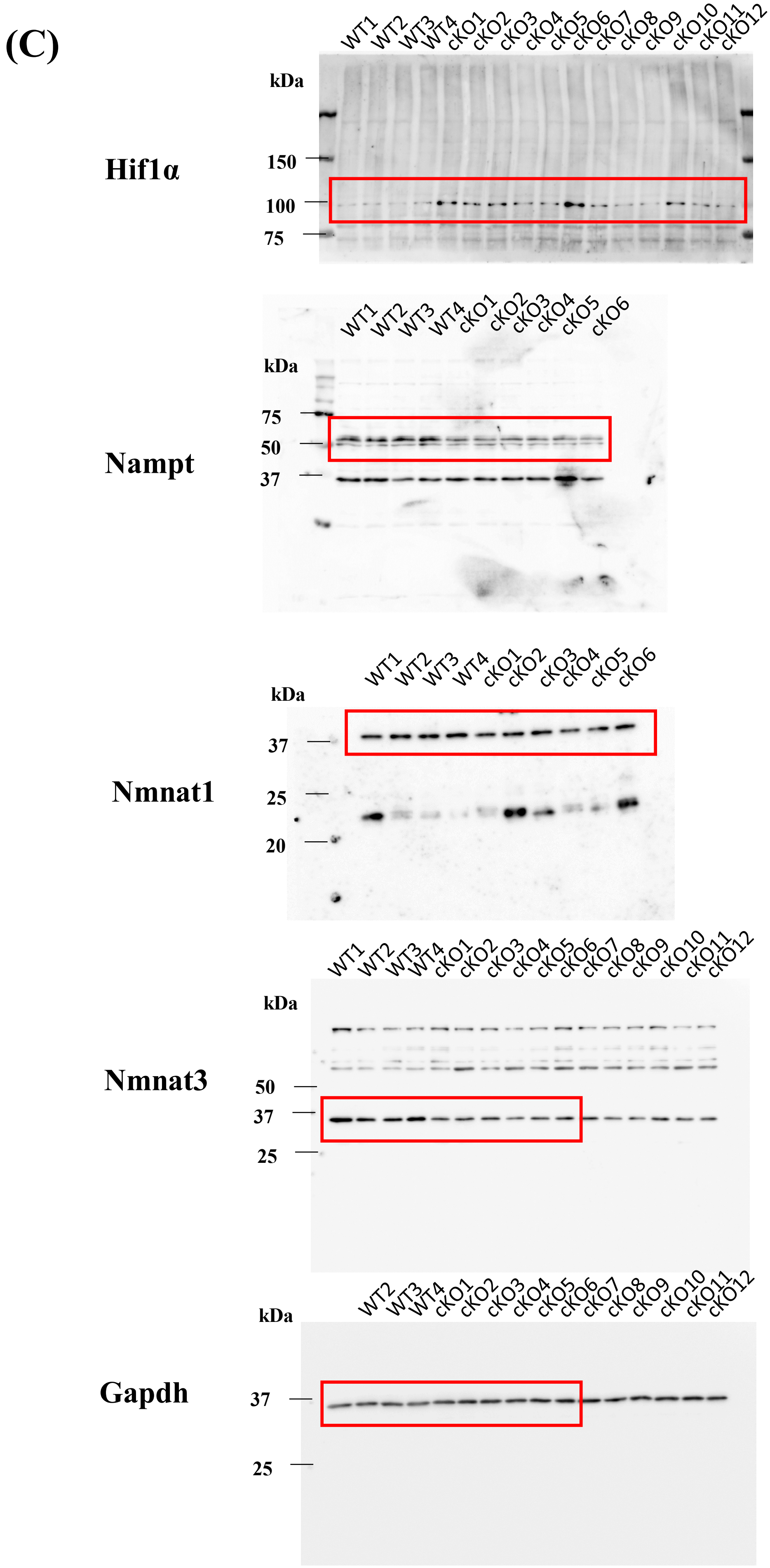

Full unedited gel for Figure 4

(E)

4-HNE

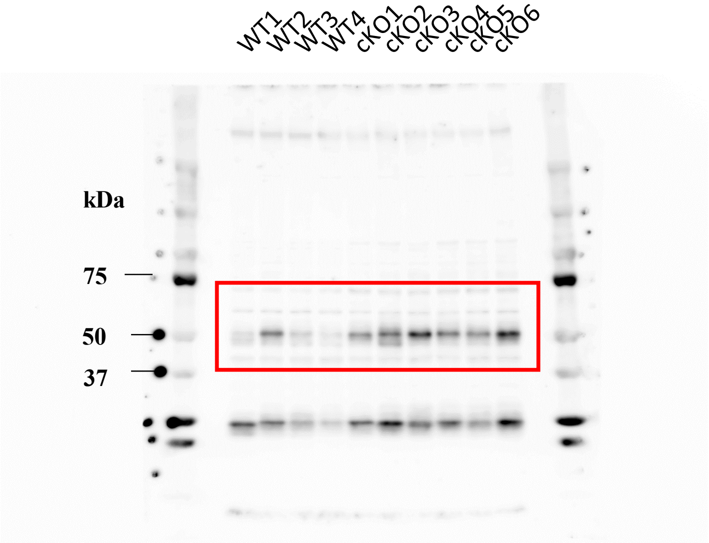

3-Nitrotyrosine

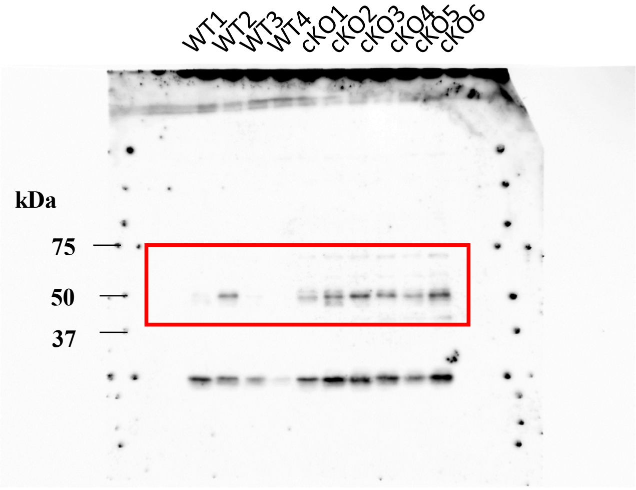

Gapdh

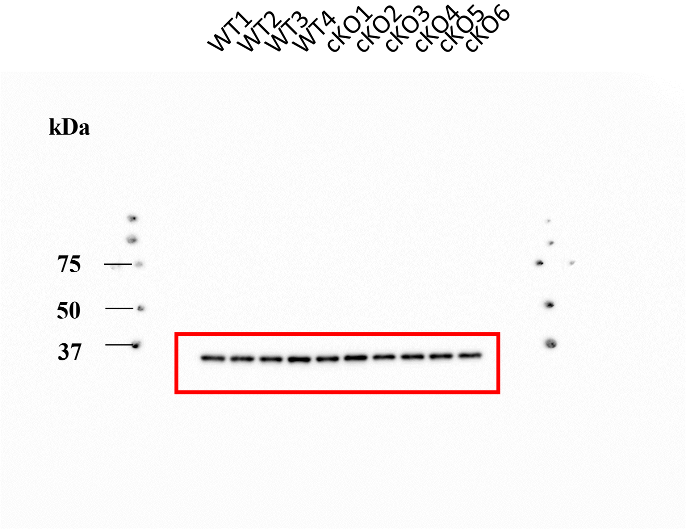

Full unedited gel for Figure 5

(A)

Lamp2

Gapdh

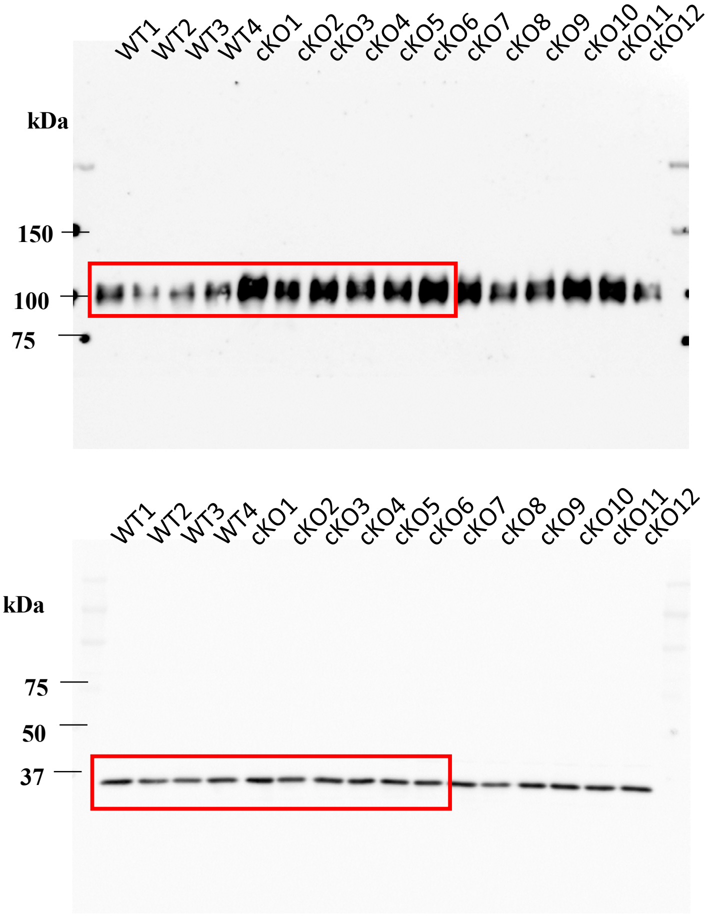

(E)

Galectin3

Vdac

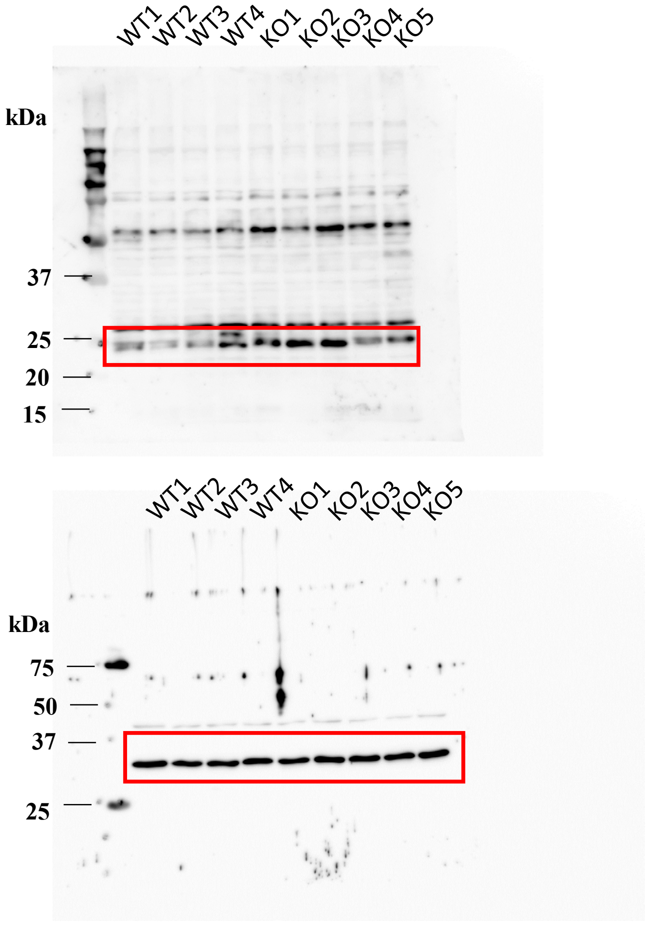

Full unedited gel for Figure 5

(F)

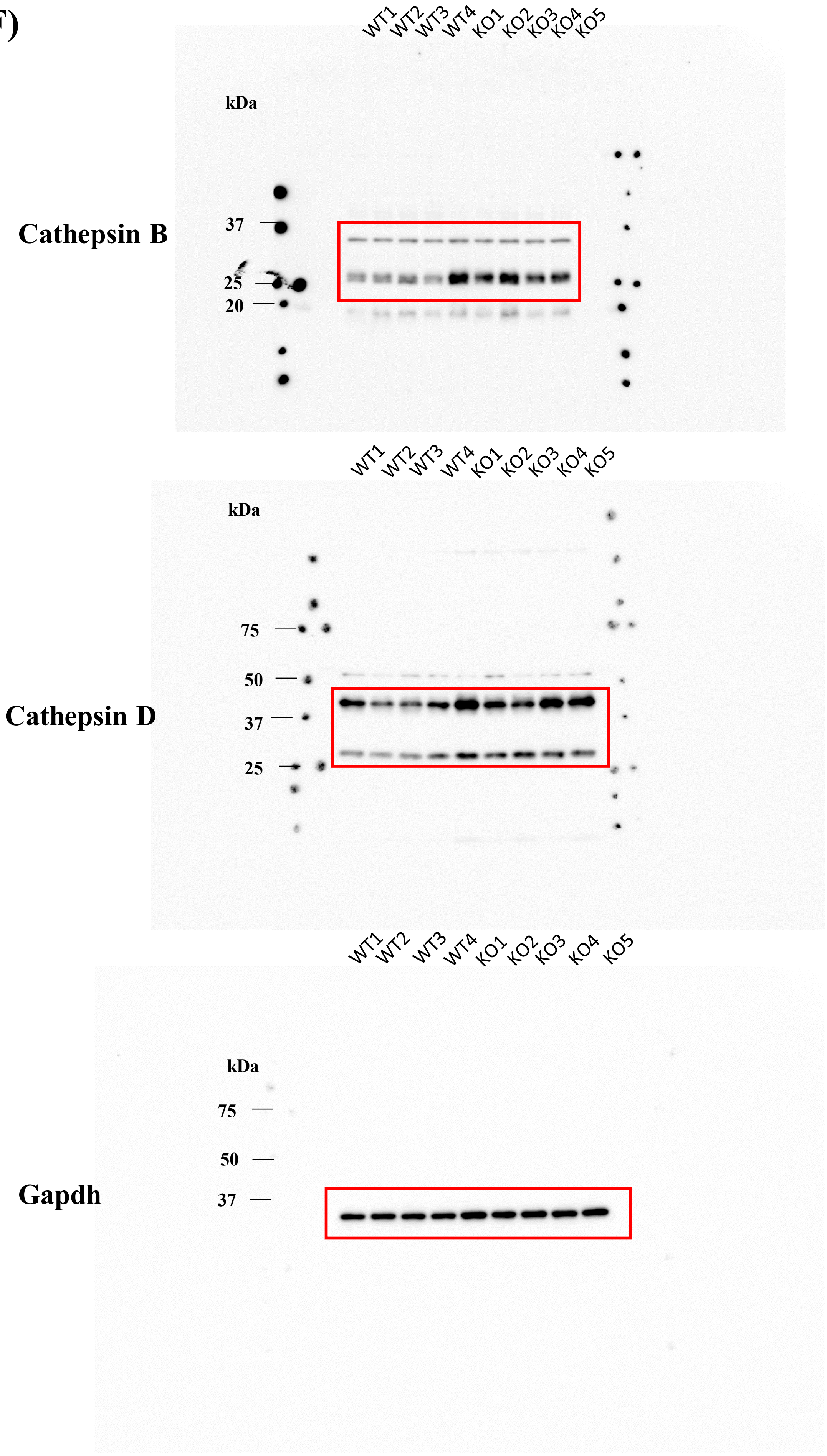

Full unedited gel for Figure 5

(G)

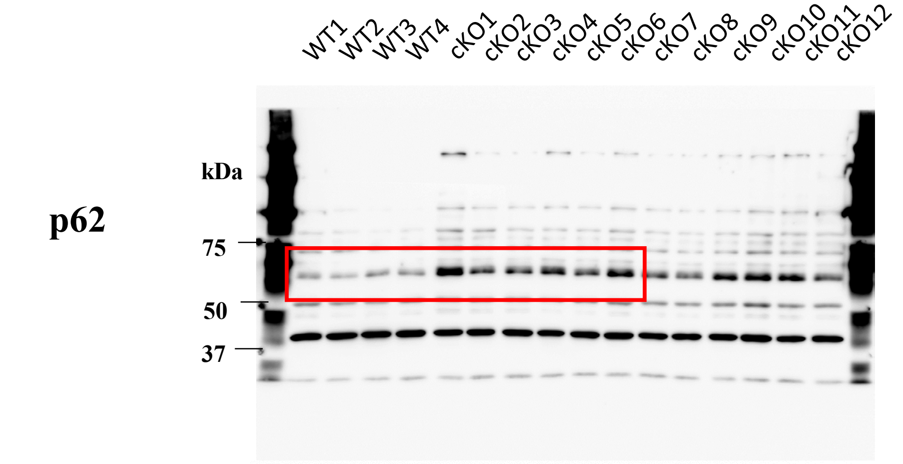

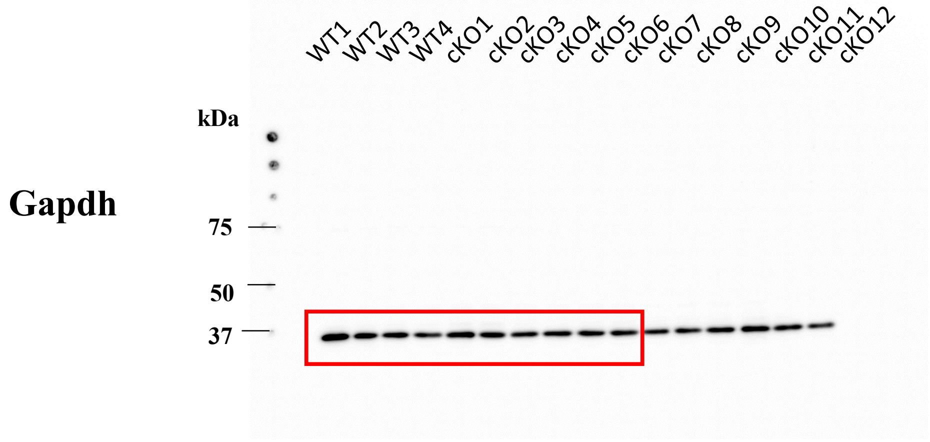

Full unedited gel for Figure 6

(B)

Gpx4

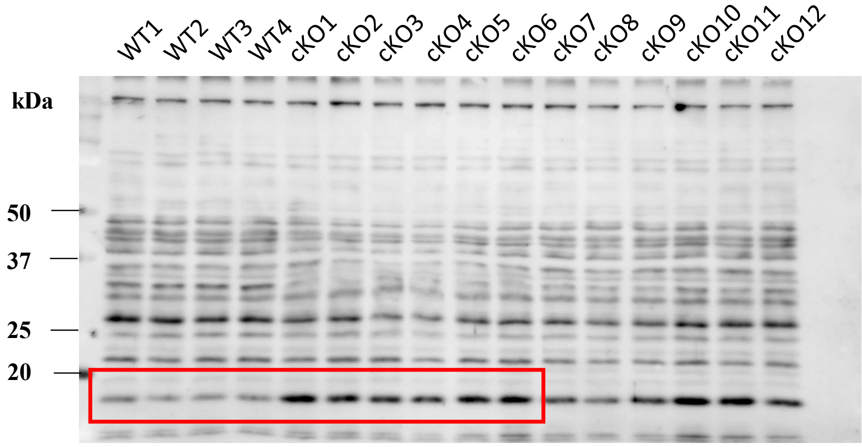

Tfrc

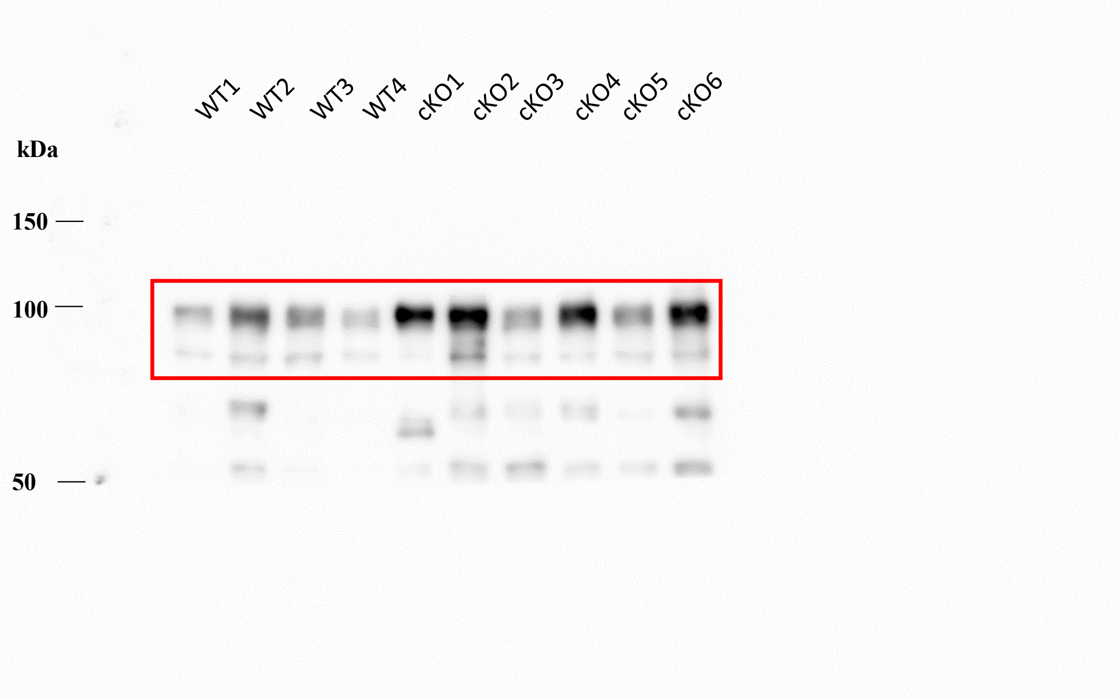

Gapdh

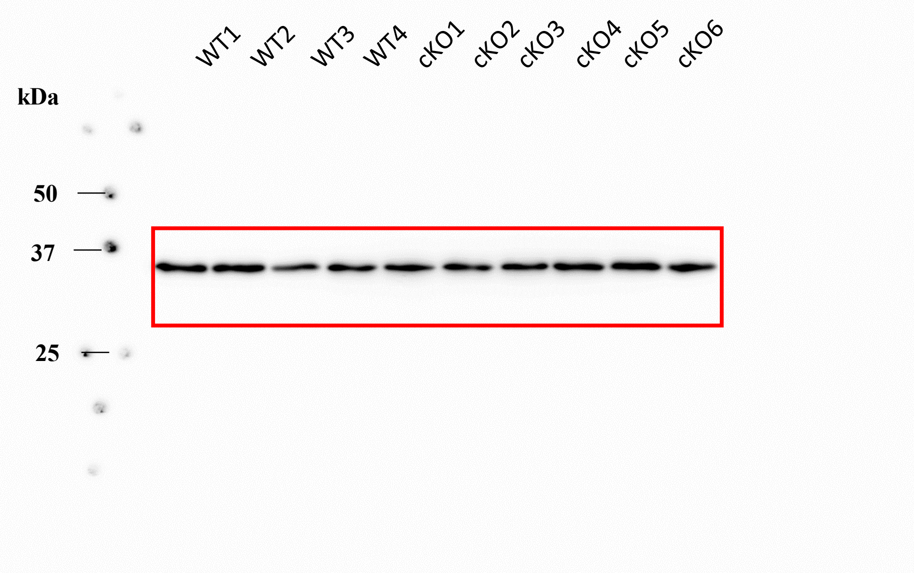

Full unedited gel for Figure 7

(B)

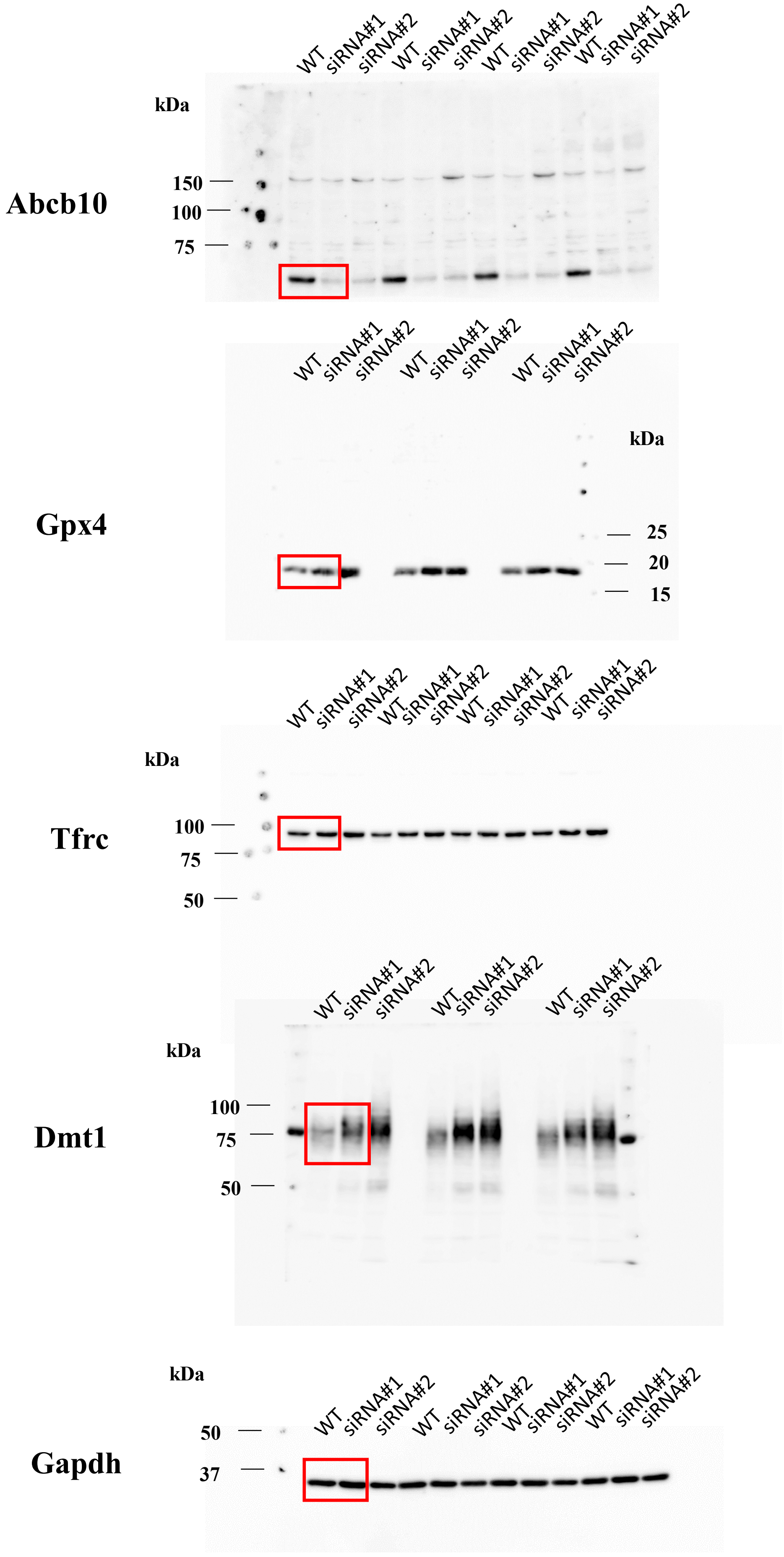

Full unedited gel for Supplementary Figure 1

(D)

Abcb10

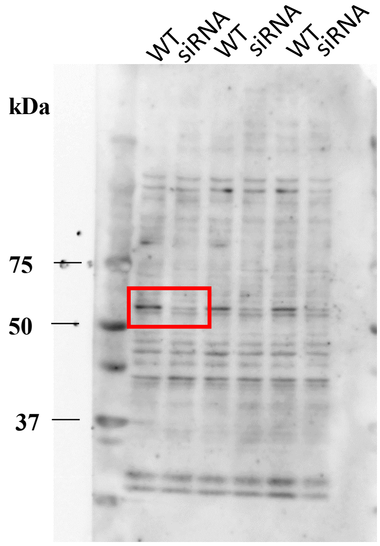

Cox Ⅰ

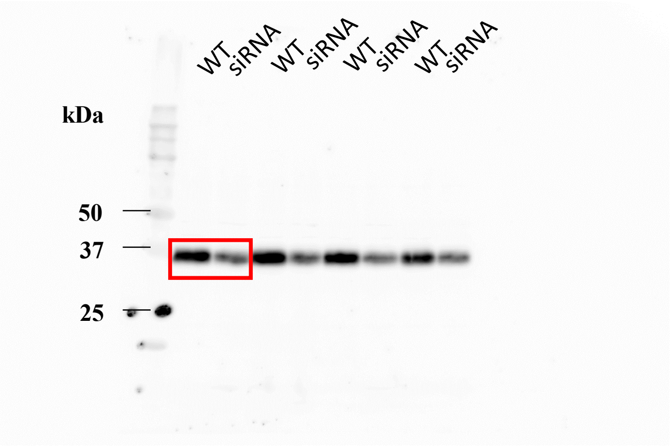

Cox Ⅱ

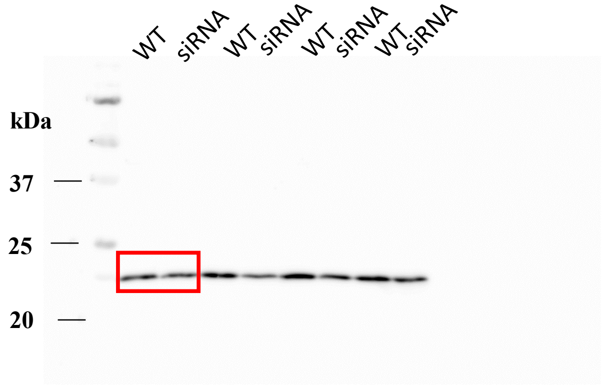

Gapdh

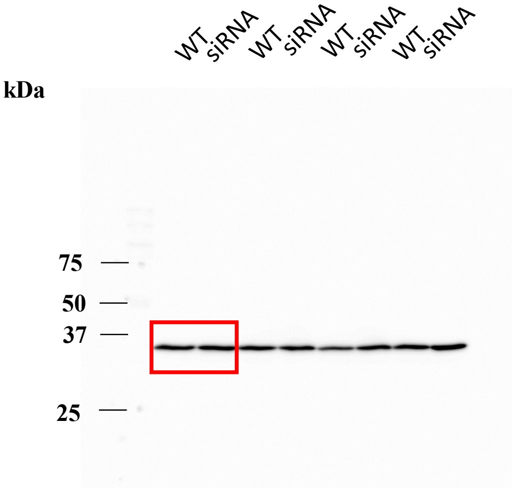

Full unedited gel for Supplementary Figure 3

(B)

Lc3

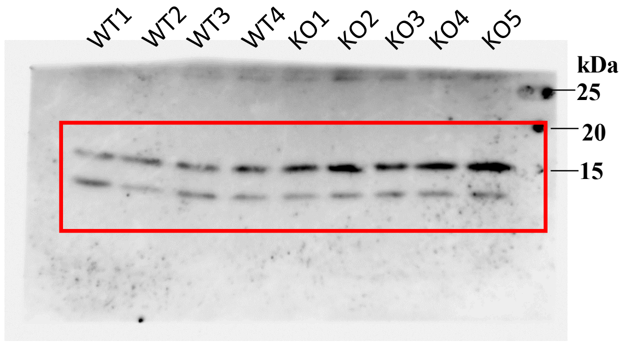

Gapdh

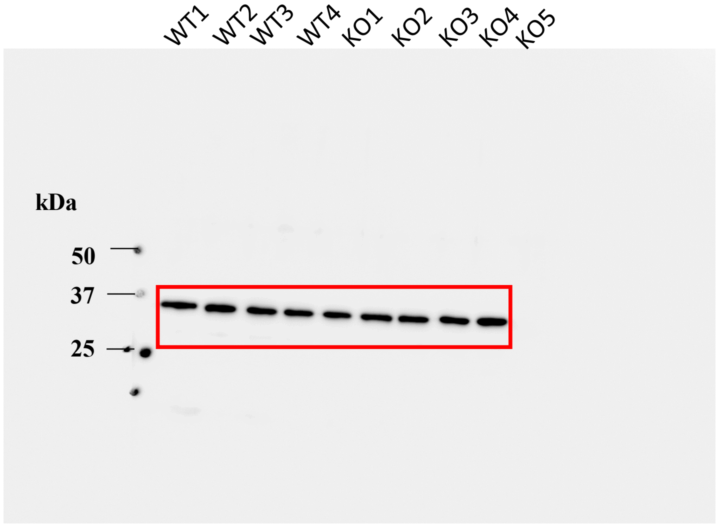

Full unedited gel for Supplementary Figure 3

(C)

Atg3

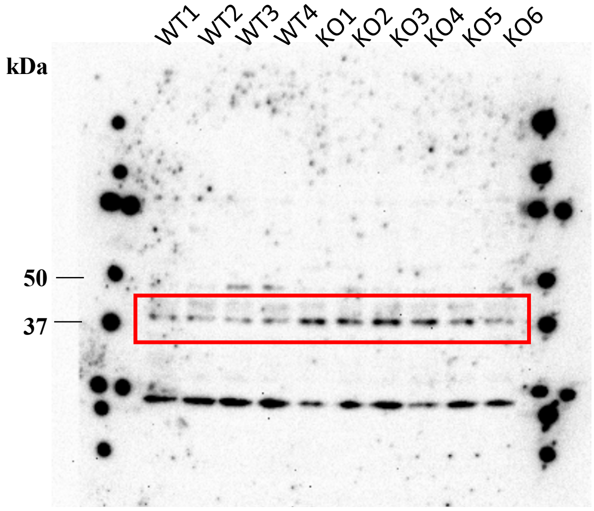

Atg5-Atg12

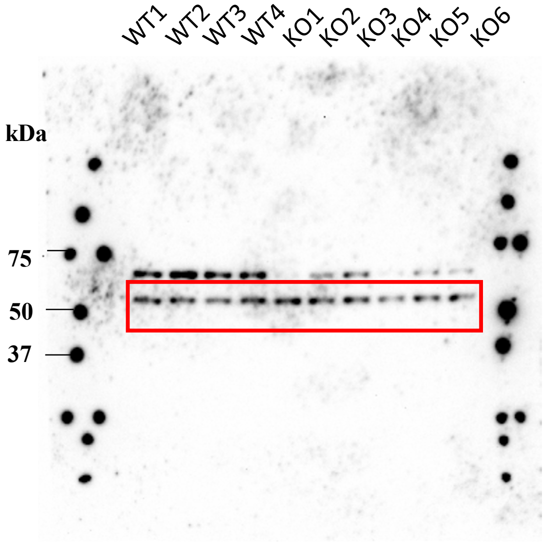

Atg7

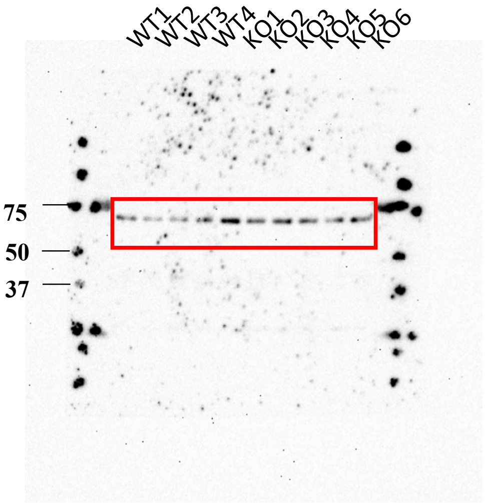

Gapdh

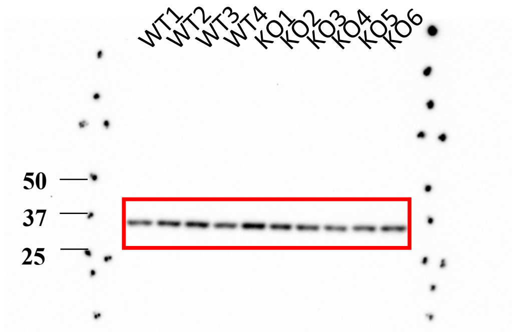

Supplement: Supplementary Figures S1-S4 and Tables S1-S2 [file BSR-2023-1992_supp.zip › BSR-2023-1992_suppd1.pdf]
