# Supplementary material for: Cardiomyocyte-specific deletion of the mitochondrial transporter Abcb10 causes cardiac dysfunction via lysosomal-mediated ferroptosis
Source: Biosci Rep. 2024 May 10;44(5):BSR20231992. doi: 10.1042/BSR20231992 (PMC11088307; doi:10.1042/BSR20231992)
Supplement: Supplementary Figures S1-S4 and Tables S1-S2 [file BSR-2023-1992_supp.zip › BSR-2023-1992_supps1.docx]

**Cardiomyocyte-specific deletion of mitochondrial transporter *Abcb10* causes cardiac dysfunction via lysosomal-mediated ferroptosis.**

Yura Do^1^, Mikako Yagi^1,2^, Haruka Hirai^2^, Kenji Miki^1^, Yukina Fukahori^2^, Daiki Setoyama^1^, Masatatsu Yamamoto^3^, Tatsuhiko, Furukawa^3^ , Yuya Kunisaki^1^, Dongchon Kang^1^, Takeshi Uchiumi^1,2*^

^1^Department of Clinical Chemistry and Laboratory Medicine, Graduate School of Medical Sciences, Kyushu University, Higashi-ku, Fukuoka 812-8582, Japan

^2^Department of Health Sciences, Graduate School of Medical Sciences, Kyushu University, Higashi-ku, Fukuoka 812-8582, Japan

^3^Department of Molecular Oncology, Graduate School Medical and Dental Sciences, Kagoshima University, 8-35-1 Sakuragaoka, Kagoshima 890-8544, Japan

^4^Department of Pathology, Graduate School Medical and Dental Sciences, Kagoshima University, 8-35-1 Sakuragaoka, Kagoshima 890-8544, Japan

**Supplementary Figure Legends**

**Supplementary Figure S1 Mitochondrial morphology in Abcb10 cKO mice hearts and ABCB10 knockdown cells and downregulation of COX I, COX II.**

(A) Relative expression of mitochondrial mRNA in Abcb10 WT, cKO hearts from 10-month-old (WT: n=4, Abcb10 cKO: n=6). Error bars means ±SD. Statistical significance was assessed by Student’s *t*-test *** *p* <0.001

(B) Electron micrographs showing mitochondria in heart tissues of Abcb10 WT and cKO mice at 10-months-old. The abnormal mitochondrial morphology in Abcb10 cKO hearts. Remnants and vacuole of the mitochondria cristae are visible within the mitochondrial matrix. Scale bar=5μm, 1μm.

(C) Mito Tracker Red staining for mitochondrial morphology in HeLa cell treated with ABCB10 siRNA for 72 hours and control. Mitochondrial morphology was graded as cells with more than 80% dot-shaped mitochondria, cells with more than 50% dot-shaped mitochondria and normal control cells. Error bars means ±SD, with >35 cells counted for each strain. Scale bars, 20 μm.

(D) Western blot analysis of COX I and COX II in HeLa cells treated with ABCB10 siRNA. Error bars means ±SD. GAPDH was used as internal control. Statistical significance was assessed by Student’s *t*-test * *p* <0.05, ** *p* <0.01, (n=3)

**Supplementary Figure S2**. **LC-MS/MS metabolomic analysis of Abcb10 cKO hearts**.

(A) Metabolite analysis of TCA cycle in the 10 months old WT and Abcb10 cKO hearts (WT: n=4, Abcb10 cKO: n=6).

(B) Metabolite analysis of amino acids in the 10 months old WT and Abcb10 cKO hearts (WT: n=4, Abcb10 cKO: n=6).

(C) Metabolite analysis of GSH and GSSG in the 10 months old WT and Abcb10 cKO hearts (WT: n=4, Abcb10 cKO: n=6).

(D) Metabolite analysis of biliverdin in hearts and in mitochondria isolated from hearts in 11 months old WT and Abcb10 cKO mice (WT: n=4, Abcb10 cKO: n=4).

In A–D, error bars are presented as mean ±SD. Statistical significance was assessed by Student’s *t*-test, **p* < 0.05, ***p* < 0.01, *** *p* <0.001.

**Supplementary Figure S3. Impaired lysosomal function and autophagy function in Abcb10 cKO hearts.**

(A) Immunostaining of p62 (green) in 10-month-old WT and Abcb10 cKO hearts. The number of ring-shaped p62 oligomers/number of DAPI-stained nuclei (blue) was quantified. Scale bar, 20 µm. Error bars are presented as means ±SD (WT, Abcb10 cKO: n = 4, 10 sheets per group).

(B) The accumulation of autophagic marker protein Lc3-Ⅰ in Abcb10 cKO hearts from 12-month-old. Gapdh was used as internal control (WT: n=4, Abcb10 cKO: n=5)

(C) Western blot analysis of the expression of autophagic proteins (Atg3, Atg5-Atg12, Atg7) in 10-month-old WT and Abcb10 cKO hearts. Gapdh was used as an internal control (WT, n = 4; Abcb10 cKO, n = 6).

In A–C, error bars are presented as mean ±SD. Statistical significance was assessed by Student’s *t*-test, **p* < 0.05, ***p* < 0.01, *** *p* <0.001.

**Supplementary Figure S4. The localization of Fe^2+^/lipid peroxides in ABCB10 knockdown cells and the cell viability with ferroptosis inducer/inhibitor in WT cardiomyocytes.**

(A) Double staining with FerroOrange and fluorescent lysosome probes LysoPrime Green in HeLa cells treated for 72 hours with ABCB10 siRNA. Scale bar, 20μm.

(B) Representative images of Mito-FerroGreen and MitoTracker Red CMXRos staining of ABCB10 siRNA-treated cells. Scale bar, 20μm.

(C) LipiRADICAL Green was co-stained with MitoTracker Red CMXRos in HeLa cells treated with ABCB10 siRNA for 72 hours. Representative colocalization pictures of lipid peroxidation in lysosome. Scale bar, 20μm.

(D) Cardiomyocytes from 9-month-old WT hearts were treated with 10μM Erastin or 5μM Ferrostatin-1(Fer-1) and cell viability assessed by live cell counting. Error bars are presented as mean ±SD. One-way ANOVA with Tukey’s multiple comparisons test, **p* < 0.05, ****p* < 0.001.

**Supplementary Table S1.** List of mouse primer used in this study

| Target | Forward | Reverse |
| --- | --- | --- |
| *Abcb10* | ttctggctgtgtccagtgtc | gcctgttcacaatgctctga |
| *Anf* | catcaccctgggcttcttcct | tgggctccaatcctgtcaatc |
| *βMHC* | atgtgccggaccttggaa | cctcgggttagctgagagatca |
| *Fgf21* | gggaggatggaacagtggta | gtcctccagcagcagttctc |
| *Gdf15* | cttgaagacttgggctggag | taagaaccaccggggtgtag |
| *Atf3* | aactggcttcctgtgcactt | ggccagctaggtcatctgag |
| *Atf4* | tcgatgctctgtttcgaatg | agaatgtaaagggggcaacc |
| *Chop* | cagaggtcacacgcacatcc | ccttgctcttcctcctcttcc |
| *Trib3* | gctgtgggattcaagccaaa | ctgtgggcctgggtactaaa |
| *Cox1* | ggtcaaccaggtgcactttt | tggggctccgattattagtg |
| *Cox2* | acgaaatcaacaaccccgta | ggcagaacgactcggttatc |
| *Cox3* | caaggccaccacactcctat | attcctgttggaggtcagca |
| *Atp6* | ccttccacaaggaactccaa | ggtagctgttggtgggctaa |
| *12s* | ccgctctacctcaccatctc | cccatttcattggctacacc |
| *16s* | gggataacagcgcaatccta | gattgctccggtctgaactc |
| *Nd1* | ggatccgagcatcttatcca | ggtggtactcccgctgtaaa |
| *Nd2* | agggatcccactgcacatag | ctcctcatgcccctatgaaa |
| *Nampt* | tacagtggccacaaattcca | caattcccgccacagtatct |
| *Nmnat1* | gaagtgggctgatcaaaagc | ccagcccgagtgatacagat |
| *Nmnat3* | tccagcagtttcagcacaac | gaggccctctagccagtctt |
| *Ptgs2* | agaaggaaatggctgcagaa | gctcggcttccagtattgag |
| *Chac1* | ataccaagttcgaggggagc | tctgtgtggcaatgacctct |
| *Ho1* | taagctggtgatggcttcct | cctgagaggtcacccaggta |
| 18S | cgcggttctattttgttggt | agtcggcatcgtttatggtc |

List of human primer used in this study.

| Target | Forward | Reverse |
| --- | --- | --- |
| ABCB10 | tgatcgtgctttctgtcctg | ctcgttaaaaggcagcttgg |
| PTGS2 | agaaggaaatggctgcagaa | gctcggcttccagtattgag |
| TFRC | aaaatccggtgtaggcacag | cctttaaatgcagggacgaa |
| DMT1 | caccggaccaggttttctta | ttgggatactgacggtgaca |
| 18S | aaacggctaccacatccaag | cctccaatggatcctcgtta |

**Supplementary Table S2.** List of antibody used in this study.

| Antibody | Reference or Source | Identifier or catalog number |
| --- | --- | --- |
| ABCB10 Polyclonal antibody | Proteintech | 14628-1-AP |
| GAPDH (14C10) Rabbit mAb | Cell Signaling | 2118 |
| Anti-4 Hydroxynonenal antibody [HNEJ-2] | abcam | ab48506 |
| Anti-3-Nitrotyrosine antibody [39B6] | abcam | ab61392 |
| Anti-MTCO1 antibody [1D6E1A8] | abcam | ab14705 |
| Anti-MTCOX3 antibody [DA5BC4] | abcam | ab110259 |
| NDUFA9 Antibody | Invitrogen | 459100 |
| SDHA Antibody | Invitrogen | 459200 |
| Anti-Ubiquinol-Cytochrome C Reductase Core ProteinⅠ antibody [16D10AD9AH5] | abcam | ab110252 |
| Complex Ⅴ alpha- | Invitrogen | 439800 |
| Purified Mouse Anti-DLP1 | BD Transduction Laboratories^TM^ | 611112 |
| Mitofusin-2 (D2D10) Rabbit mAb | Cell Signaling | 9482 |
| Purified Mouse Anti-OPA1 | BD Transduction Laboratories^TM^ | 612606 |
| Anti-Hif1 alpha antibody | abcam | ab110333 |
| PBEF/Visfatin/NAMPT Antibody | NOVUS | NB100-594 |
| NMNAT-1 (B-7) antibody | Santa Cruz | sc-271557 |
| NMNAT-3 (D-10) antibody | Santa Cruz | sc-390433 |
| LAMP2 antibody [GL2A7] | abcam | ab13524 |
| Cathepsin B (D1C7Y) XP Rabbit mAb | Cell Signaling | 31718 |
| Anti-Cathepsin D Antibody [EPR3057Y] | abcam | ab75852 |
| Anti-Galectin3 antibody [A3A12] | abcam | ab2785 |
| VDAC | Yagi et al (2012) |  |
| LC3A/B (D3U4C)XP Rabbit mAb | Cell Signaling | 12741 |
| Anti-p62(SQSTM1) pAb | MBL | PM045 |
| GPX4 antibody | Cell Signaling | 52455 |
| Transferrin Receptor Monoclonal Antibody (H68.4) | Invitrogen | #13-6800 |
| DMT1/SLC11A2(D3V8G) Rabbit mAb | Cell Signaling | 15083 |
