# Supplementary material for: Cardiomyocyte-specific deletion of the mitochondrial transporter Abcb10 causes cardiac dysfunction via lysosomal-mediated ferroptosis
Source: Biosci Rep. 2024 May 10;44(5):BSR20231992. doi: 10.1042/BSR20231992 (PMC11088307; doi:10.1042/BSR20231992)

Supplementary Figure 1

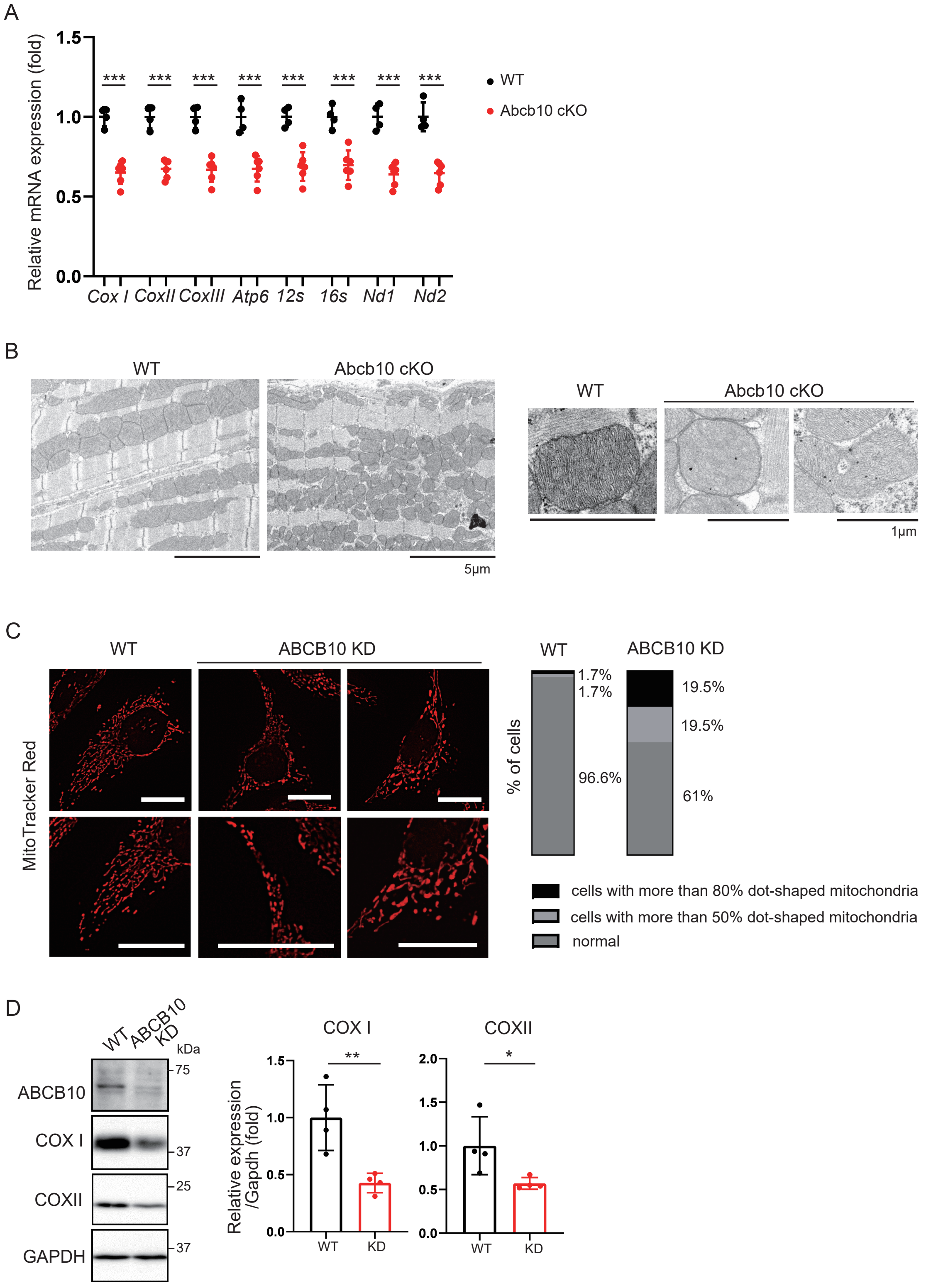

Supplementary Figure 2

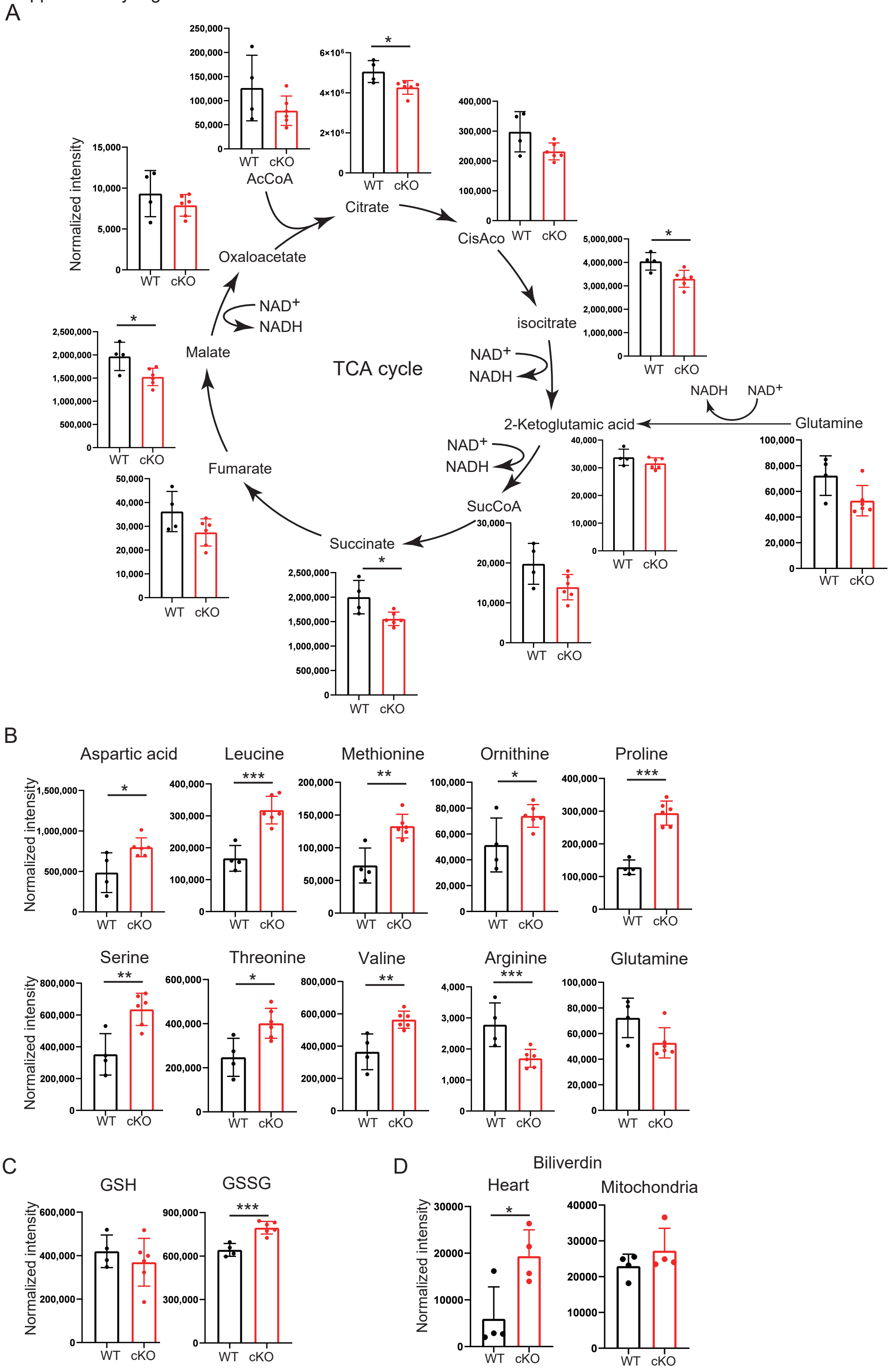

Supplementary Figure 3

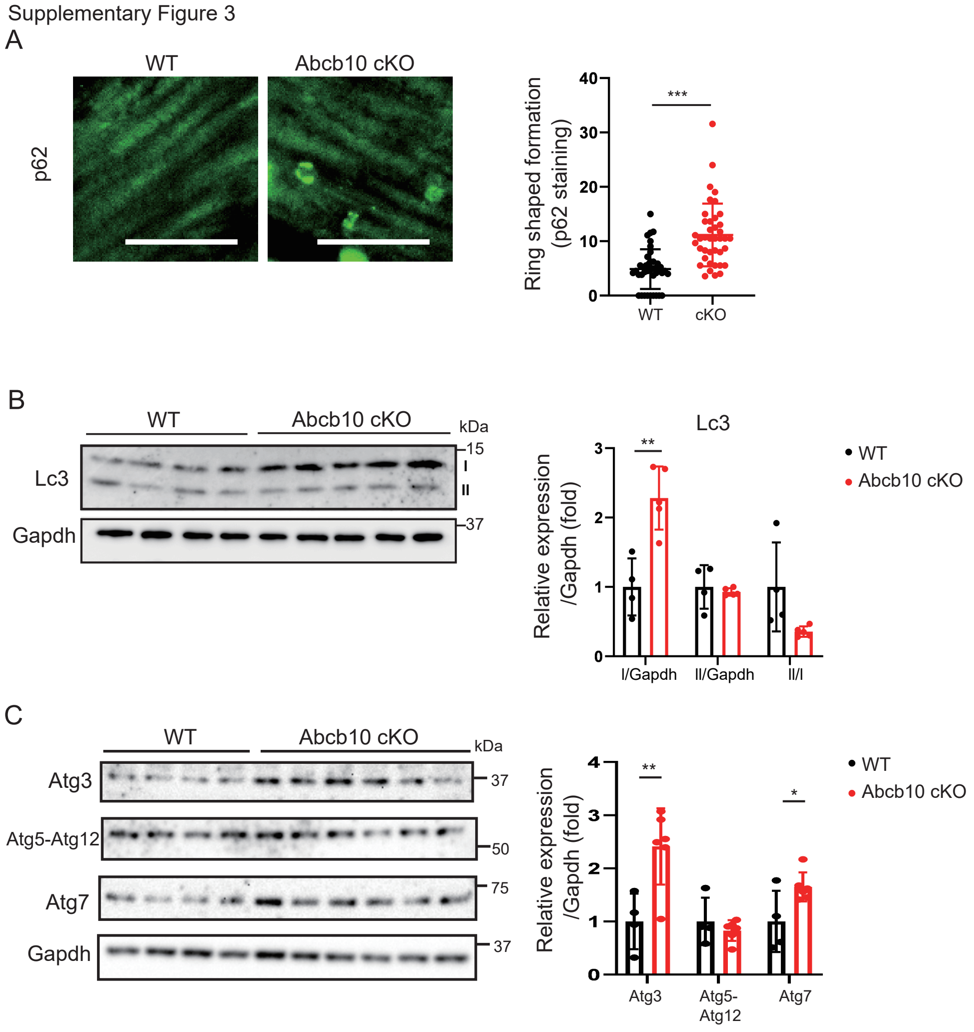

Supplementary Figure 4

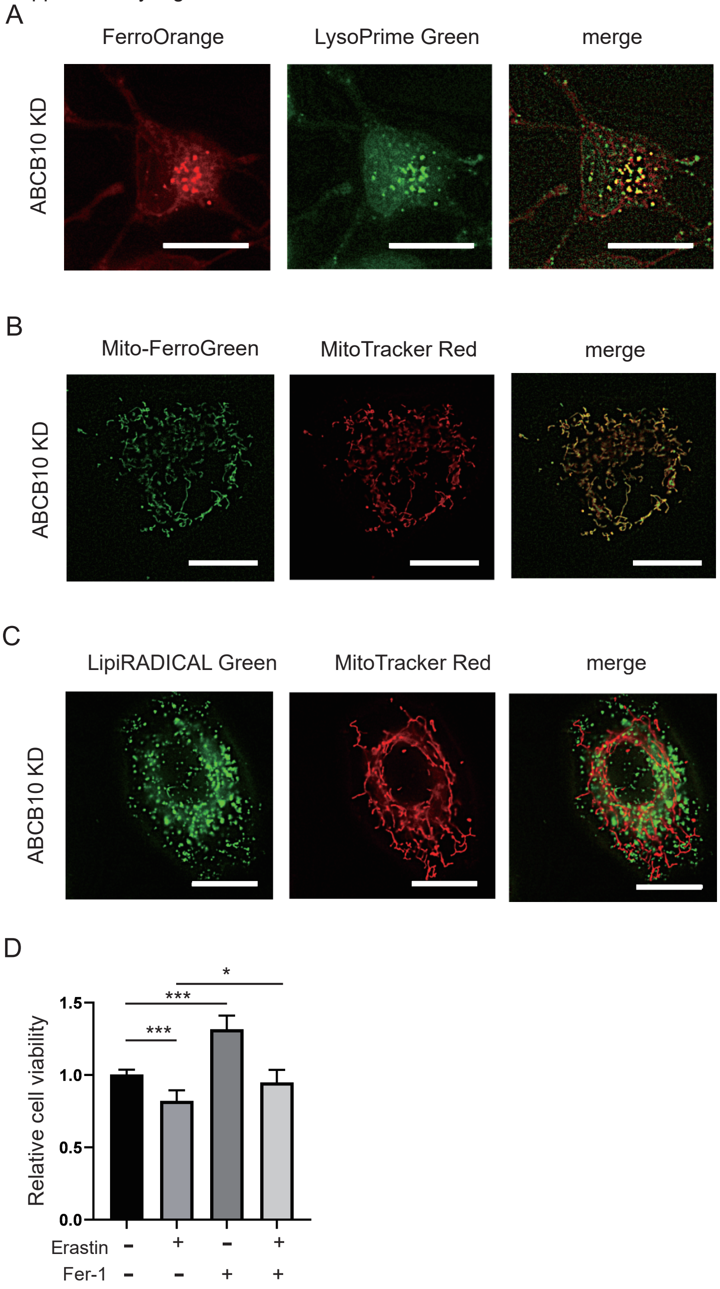

Supplement: Supplementary Figures S1-S4 and Tables S1-S2 [file BSR-2023-1992_supp.zip › BSR-2023-1992_supps2.pdf]
